# Colossal Reversible Barocaloric Effects in a Plastic Crystal Mediated by Lattice Vibrations and Ion Diffusion

**DOI:** 10.1002/advs.202306488

**Published:** 2024-05-05

**Authors:** Ming Zeng, Carlos Escorihuela‐Sayalero, Tamio Ikeshoji, Shigeyuki Takagi, Sangryun Kim, Shin‐ichi Orimo, María Barrio, Josep‐Lluís Tamarit, Pol Lloveras, Claudio Cazorla, Kartik Sau

**Affiliations:** ^1^ Grup de Caracterizació de Materials, Departament de Física EEBE and Barcelona Research Center in Multiscale Science and Engineering Universitat Politècnica de Catalunya Av. Eduard Maristany 10‐14 Barcelona 08019 Catalonia Spain; ^2^ Mathematics for Advanced Materials Open Innovation Laboratory (MathAM‐OIL) National Institute of Advanced Industrial Science and Technology (AIST) c/o Advanced Institute for Materials Research (AIMR), Tohoku University Sendai 980‐8577 Japan; ^3^ Institute for Materials Research (IMR) Tohoku University Sendai 980‐8577 Japan; ^4^ Graduate School of Energy Convergence Gwangju Institute of Science and Technology (GIST) 123 Cheomdangwagi‐ro, Buk‐gu Gwangju 61005 Republic of Korea; ^5^ Advanced Institute for Materials Research (AIMR) Tohoku University Sendai 980‐8577 Japan

**Keywords:** barocaloric effects, lithium diffusion, molecular dynamics simulations, orientational order–disorder phase transition, solid‐state refrigeration

## Abstract

Solid‐state methods for cooling and heating promise a sustainable alternative to current compression cycles of greenhouse gases and inefficient fuel‐burning heaters. Barocaloric effects (BCE) driven by hydrostatic pressure (*p*) are especially encouraging in terms of large adiabatic temperature changes (|Δ*T*| ≈ 10 K) and isothermal entropy changes (|Δ*S*| ≈ 100 J K^−1^ kg^−1^). However, BCE typically require large pressure shifts due to irreversibility issues, and sizeable |Δ*T*| and |Δ*S*| seldom are realized in a same material. Here, the existence of colossal and reversible BCE in LiCB_11_H_12_ is demonstrated near its order‐disorder phase transition at ≈380 K. Specifically, for Δ*p* ≈ 0.23 (0.10) GPa, |Δ*S*_rev_| = 280 (200) J K^−1^ kg^−1^ and |Δ*T*_rev_| = 32 (10) K are measured, which individually rival with state‐of‐the‐art BCE figures. Furthermore, pressure shifts of the order of 0.1 GPa yield huge reversible barocaloric strengths of ≈2 J K^−1^ kg^−1^ MPa^−1^. Molecular dynamics simulations are performed to quantify the role of lattice vibrations, molecular reorientations, and ion diffusion on the disclosed BCE. Interestingly, lattice vibrations are found to contribute the most to |Δ*S*| while the diffusion of lithium ions, despite adding up only slightly to the entropy change, is crucial in enabling the molecular order–disorder phase transition.

## Graphical abstract

The presence of colossal and reversible barocaloric effects in LiCB_11_H_12_, a renowned solid electrolyte, is evidenced near its order–disorder phase transition. It is observed that lattice vibrations primarily contribute to these barocaloric effects. Additionally, although the diffusion of lithium ions only marginally contributes to the accompanying entropy change, it plays a crucial role in facilitating the molecular order–disorder phase transition.

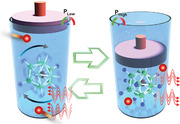

## 1 Introduction

Solid‐state methods for cooling and heating are energy efficient and ecologically friendly techniques with potential for solving the environmental problems posed by conventional refrigeration and heat pump technologies relying on compression cycles of greenhouse gases and inefficient traditional fuel‐burning heaters.^[^ ^1^ ^]^ For instance, gas boilers typically present a coefficient of performance (COP) below 2, even when accounting for heat recovery, while heat pumps may exhibit a COP ranging from 3 to 5.^[^ ^2^, ^3^, ^4^ ^]^ Under moderate magnetic, electric, or mechanical field variations, auspicious caloric materials experience large adiabatic temperature variations (|Δ*T*| ≈ 1–10 K) as a result of phase transformations entailing large isothermal entropy changes (|Δ*S*| ≈ 10–100 J K^−1^ kg^−1^).^[^ ^5^, ^6^ ^]^ Gas adsorption processes inducing large structural changes, designated as breathing transitions, have also been proposed as an innovative strategy to drive very large thermal changes in porous materials by means of hydrostatic pressure.^[^ ^7^, ^8^ ^]^ Solid‐state cooling and heat pumping capitalize on such caloric effects for engineering refrigeration and heating cycles, presenting, in some cases, COPs exceeding 10.^[^ ^9^, ^10^, ^11^ ^]^

From a practical point of view, large and reversible |Δ*T*| and |Δ*S*| are both necessary for achieving rapid and efficient thermal devices under recursive application and removal of the driving fields. The maximum amount of heat that can be removed from a heat source per cycle is dictated by |Δ*S*| while the rate at which the heat extracted from the heat source is transferred to a heat sink is governed by |Δ*T*|. In terms of largest |Δ*T*| and |Δ*S*|, mechanocaloric effects induced by uniaxial stress (elastocaloric effects) and hydrostatic pressure (barocaloric effects –BCE–) are among the most promising.^[^ ^12^, ^13^, ^14^, ^15^ ^]^

Recently, colossal and reversible BCE (|Δ*S*_rev_| ⩾ 100 J K^−1^ kg^−1^) have been measured in several families of materials displaying order–disorder phase transitions under pressure shifts of the order of 0.1 GPa.^[^ ^16^, ^17^, ^18^, ^19^, ^20^, ^21^, ^22^, ^23^, ^24^ ^]^ On one hand, there are plastic crystals like neopentane derivatives,^[^ ^16^, ^17^, ^18^ ^]^ adamantane derivatives,^[^ ^19^, ^24^ ^]^ and carboranes^[^ ^20^ ^]^ in which the underlying phase transitions involve molecular orientational disorder stabilized under increasing temperature. On the other hand, there are polymers (e.g., acetoxy silicone rubber),^[^ ^21^ ^]^ hybrid organic–inorganic layered perovskites (e.g., [C_10_H_21_NH_3_]_2_MnCl_4_)^[^ ^22^, ^23^ ^]^ and salts ([DBA][BF_4_] and dialkylammonium halides)^[^ ^25^, ^26^ ^]^ in which the accompanying phase transformations entail significant atomic rearrangements in the organic components. Another family of disordered materials presenting also great barocaloric promise are solid electrolytes (e.g., AgI, Li_3_N, and Cu_2_Se),^[^ ^27^, ^28^, ^29^, ^30^ ^]^ although in this latter case the experimentally reported |Δ*S*_rev_| fall slightly below the colossal threshold value of 100 J K^−1^ kg^−1^.^[^ ^27^ ^]^

In spite of these recent developments, finding barocaloric materials with well‐balanced and suitable features for developing thermal applications, namely, |Δ*T*_rev_| ⩾ 20 K and |Δ*S*_rev_| ⩾ 100 J K^−1^ kg^−1^ driven by Δ*p* ≲ 0.1 GPa, is proving very difficult. These indicative criteria respond to the facts that i) the material must be capable of achieving higher temperatures than the hot end and lower temperatures than the cold end, typically separated by 15 to 20 degrees for air conditioning and refrigeration, ii) the |Δ*S*_rev_| values for current fluid refrigerants are approximately 500 J K^−1^ kg^−1^,^[^ ^31^ ^]^ making a lower threshold of 100 J K^−1^ kg^−1^ a reasonable choice for initial material selection in proof‐of‐concept studies, and iii) the maximum pressure change for devices is stated as 0.1 GPa, given the current state of the art in solid barocaloric materials and the availability of off‐the‐shelf compressors.^[^ ^11^ ^]^

From the hundreds of barocaloric materials known to date,^[^ ^15^ ^]^ to the best of our knowledge only five fulfill the threshold conditions specified above, namely, the spin‐crossover complex Fe_3_(bntrz)_6_(tcnset)_6_ (|Δ*T*_rev_| = 35 K and |Δ*S*_rev_| = 120 J K^−1^ kg^−1^ for Δ*p* = 0.26 GPa),^[^ ^32^ ^]^ the layered hybrid perovskite [C_10_H_21_NH_3_]_2_MnCl_4_ (|Δ*T*_rev_| = 27 K and |Δ*S*_rev_| = 250 J K^−1^ kg^−1^ for Δ*p* = 0.19 GPa),^[^ ^22^, ^23^ ^]^ the plastic crystal 1‐Br‐adamantane (|Δ*T*_rev_| = 20 K and |Δ*S*_rev_| = 120 J K^−1^ kg^−1^ for Δ*p* = 0.10 GPa),^[^ ^19^ ^]^ the elastomer acetoxy silicone (|Δ*T*_rev_| = 22 K and |Δ*S*_rev_| = 182 J K^−1^ kg^−1^ for Δ*p* = 0.17 GPa),^[^ ^21^ ^]^ and the salt di‐*n*‐butylammonium tetrafluoroborate (|Δ*T*_rev_| = 17 K and |Δ*S*_rev_| = 238 J K^−1^ kg^−1^ for Δ*p* = 0.10 GPa).^[^ ^25^ ^]^ Moreover, studies addressing a fundamental and quantitative understanding of the atomistic mechanisms that bring on such colossal BCE are very scarce,^[^ ^33^, ^34^, ^35^, ^36^ ^]^ thus hindering the rational design of disordered materials with enhanced barocaloric performances.

**Figure 1:**
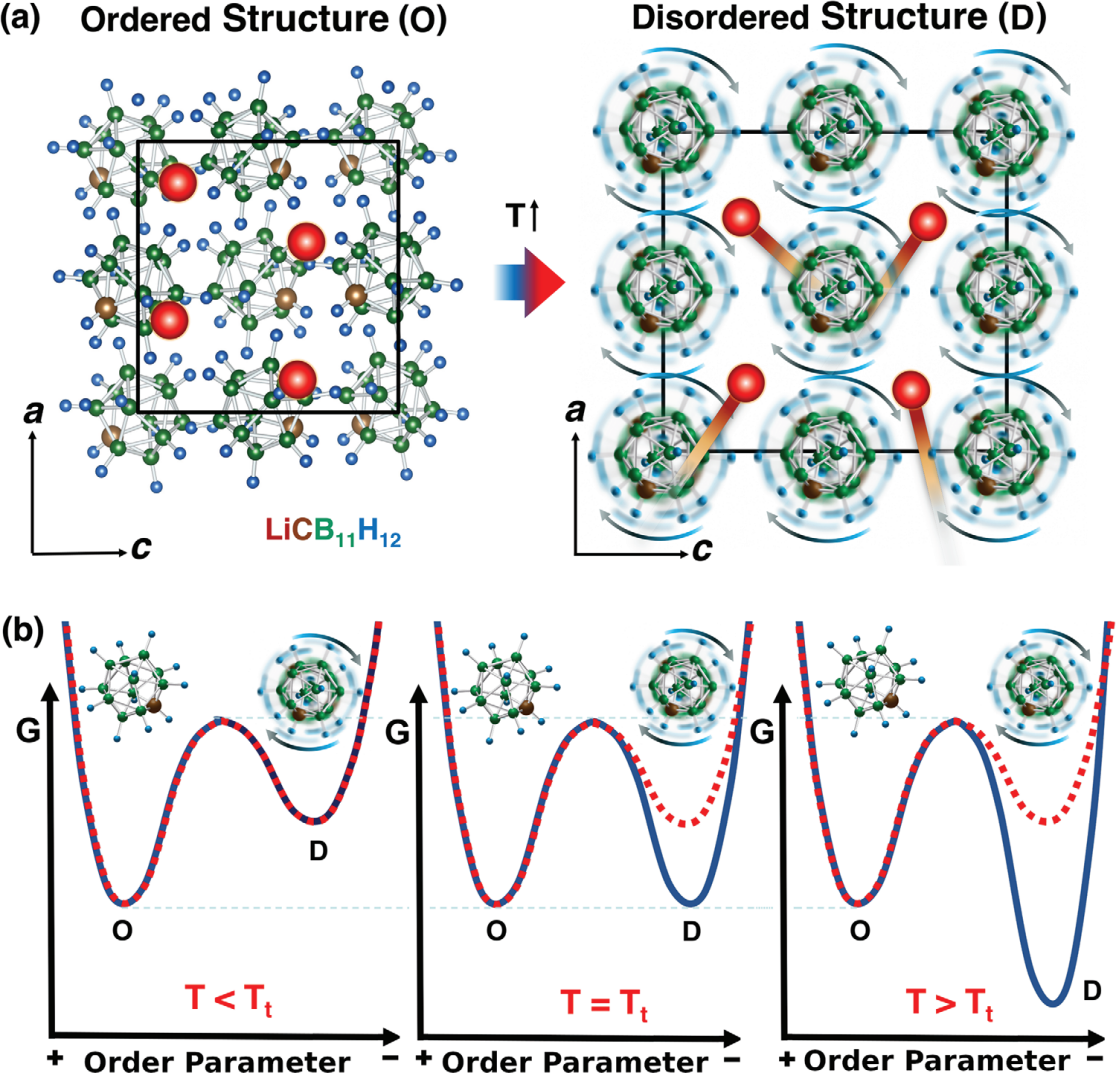
Sketch of the order–disorder phase transition occurring in LiCB_11_H_12_ upon increasing temperature. a) Ball‐stick representation of the low‐*T* ordered (O) and high‐*T* disordered (D) phases. Lithium, carbon, boron, and hydrogen atoms are represented with red, brown, green, and blue spheres, respectively. In the high‐*T* phase, the Li^+^ cations diffuse throughout the crystalline matrix while the [CB_11_H_12_]^−^ anions reorient disorderly^[^ ^37^ ^]^; the volume increases significantly during the *T*‐induced phase transition. b) Outline of the order–disorder phase transition in terms of Gibbs free energies. The red dotted lines represent internal energies and the blue solid lines Gibbs free energies; *T*_*t*_ denotes the phase transition temperature. The “Order parameter” in the *x*‐axis is of mixed molecular orientational and ionic diffusive characters.

In this work, we experimentally and theoretically demonstrate the existence of colossal and reversible BCE in the monocarba‐*closo*‐dodecaborate LiCB_11_H_12_ (LCBH) near its order–disorder phase transition occurring at *T*_*t*_ ≈ 380 K.^[^ ^37^ ^]^ LCBH is a well‐known solid electrolyte^[^ ^38^, ^39^ ^]^ in which at temperatures above *T*_*t*_ the lithium cations are highly mobile and the molecular anions [CB_11_H_12_]^−^ reorient disorderly^[^ ^40^, ^41^ ^]^ (**Figure** **1**). Thus, LCBH combines phase‐transition features of both plastic crystals and superionic compounds, two families of materials for which colossal and giant BCE, respectively, have been previously reported.^[^ ^16^, ^17^, ^18^, ^27^ ^]^ The effects of hydrostatic pressure on the order–disorder solid–solid phase transition of LCBH, however, have remained largely unexplored.

In particular, we measured colossal values of |Δ*T*_rev_| = 32 K and |Δ*S*_rev_| = 280 JK^−1^kg^−1^ for a pressure shift of 0.23 GPa, and large and reversible barocaloric strengths of ≈2 J K^−1^ kg^−1^ MPa^−1^ over a wide temperature interval of several tens of degrees. Likewise, for a smaller pressure shift of 0.10 GPa assuring values of |Δ*S*_rev_| = 200 J K^−1^ kg^−1^ and |Δ*T*_rev_| = 10 K were obtained. Atomistic molecular dynamics simulations were performed to reveal key phase transition mechanisms and quantify the role played by the vibrational, molecular orientational, and ion diffusive degrees of freedom on the disclosed BCE. Very interestingly, the contribution of the lattice vibrations to Δ*S* was found to be the dominant at all pressures, instead of the typically assumed one resulting from molecular reorientational motion.^[^ ^33^, ^34^, ^35^ ^]^

Our results provide new valuable insights into the physical behavior of superionic plastic crystals and offers original functionalities based on the use of hydrostatic pressure, namely, colossal BCE. Similar barocaloric phenomena to those reported here for LCBH could also exist in other akin closo‐borate materials like NaCB_11_B_12_,^[^ ^37^, ^40^ ^]^ KCB_11_B_12_,^[^ ^42^ ^]^ and LiCB_9_H_10_.^[^ ^43^, ^44^ ^]^

## 2 Results and Discussion

### 2.1 LiCB_11_H_12_ General Properties

In a recent X‐ray powder diffraction study,^[^ ^37^ ^]^ it has been shown that at room temperature LiCB_11_H_12_ (LCBH) presents an ordered orthorhombic structure (space group *Pca*2_1_) in which the Li^+^ cations reside near trigonal‐planar sites surrounded by molecular [CB_11_H_12_]^−^ anions arranged in a cubic sublattice. An order–disorder phase transition occurs at *T*_*t*_ ≈ 380 K that stabilizes a disordered phase in which the Li^+^ cations are highly mobile and the molecular anions present fast reorientational motion (Figure 1a). At normal pressure, the lithium ion conductivity measured just above *T*_*t*_ exceeds values of 0.1 S cm^−1^^[^ ^37^ ^]^ and the reorientational motion of the molecular anions can reach frequencies of 10^11^ s^−1^.^[^ ^37^, ^45^ ^]^ Meanwhile, the *T*‐induced order–disorder phase transition is accompanied by a huge volume increase of ≈10%^[^ ^45^ ^]^ that, based on the Clausius‐Clapeyron (CC) equation $\Delta S_{t}=\Delta V_{t}\frac{dp}{dT}$, suggests great barocaloric potential. A general description of LCBH in terms of practical aspects like cost, processability and scalability can be found in the Supporting Information.

The described order–disorder phase transition can be qualitatively understood in terms of the Gibbs free energy difference between the high‐*T* disordered (D) and low‐*T* ordered (O) phases, Δ*G* ≡ *G* ^*D*^ − $G^{O}$ (Figure 1b). This free energy difference consists of an internal energy (Δ*E*), entropy (−*T*Δ*S*), and volume (*p*Δ*V*) terms. The internal energy term remains large and positive, thereby discouraging the stabilization of the disordered phase, much like the volume term (Δ*V* = *V* ^*D*^ − $V^{O}$ > 0). Consequently, the order–disorder phase transition in LCBH is primarily driven by the change in entropy, Δ*S*, which is notably significant due to the presence of ionic and molecular orientational disorder above *T*_*t*_. For instance, at a pressure of 0.1 GPa, and based in our molecular dynamics simulations (see below), we estimated Δ*E* = 109, *p*Δ*V* = 8, and −*T*Δ*S* = −117 kJ kg^−1^.

### 2.2 Experimental Barocaloric Results

**Figure 2:**
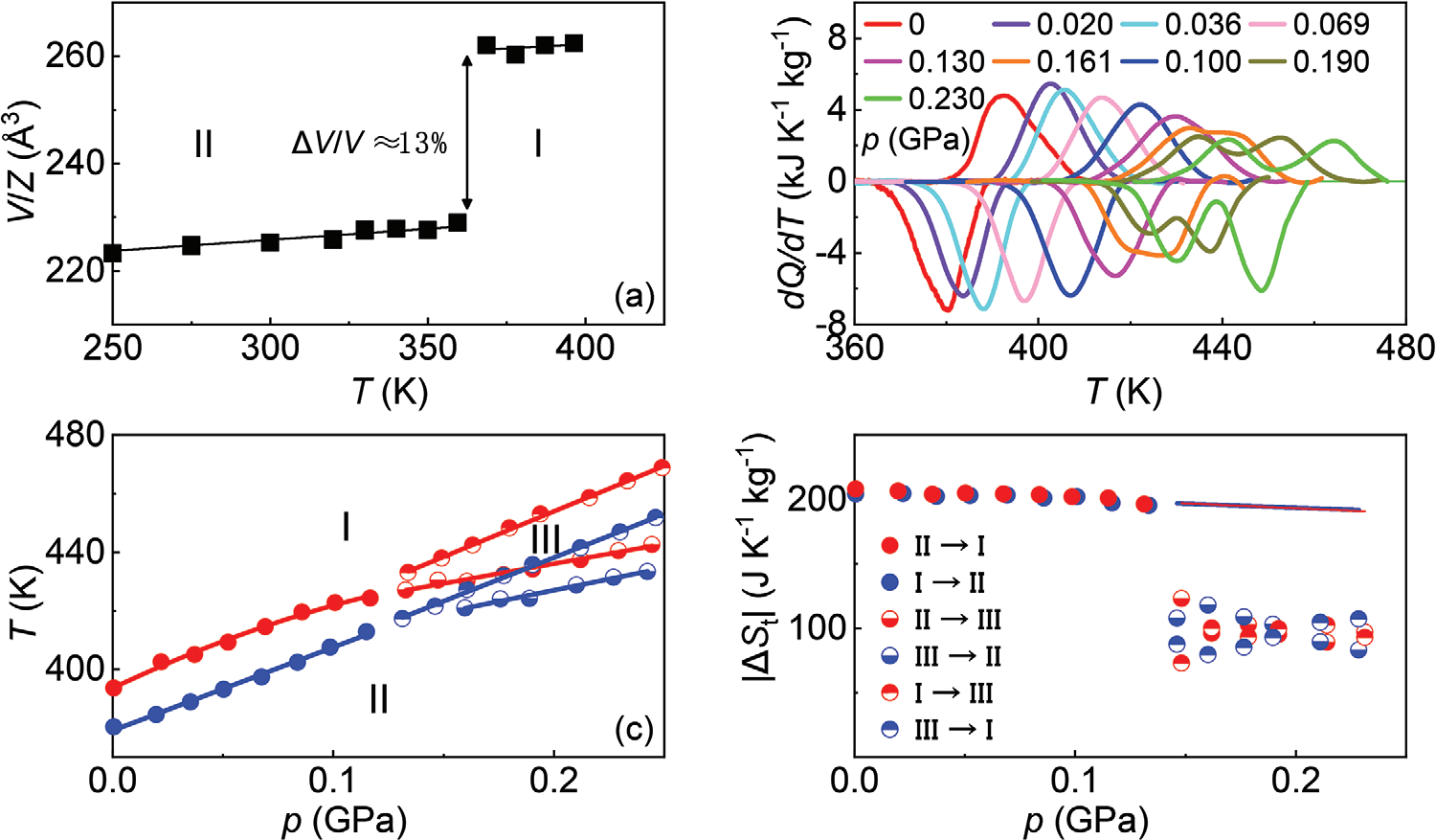
Experimental phase diagram of bulk LiCB_11_H_12_ and corresponding phase transition entropy changes. a) Volume per formula unit measured as a function of temperature at normal pressure. b) Isobaric heat flow data expressed as a function of applied pressure and temperature; data collected during heating (cooling) are represented in the positive (negative) y‐axis. c) Pressure and temperature phase diagram; transition temperatures are determined from the peaks in panel (b). d) Phase transition entropy changes as a function of pressure and transition path. Δ*S*_t_ remains practically constant from atmospheric pressure all the way up to the triple point. At *p* ≃ 0.13 GPa, Δ*S*_II → I_ ≈ Δ*S*_II → III_ + Δ*S*_III → I_, while above the triple point Δ*S*_II → III_ ≈ Δ*S*_III → I_. Straight lines at pressures above the triple point are linear fits to Δ*S*_II → III_ + Δ*S*_III → I_.

Conventional X‐ray powder diffraction experiments performed at normal pressure and under varying temperature confirmed the expected structures of the low‐*T* and high‐*T* phases (orthorhombic and cubic symmetry, respectively, Figure S1, Supporting Information). Pattern matching analysis of the obtained data yielded the temperature‐dependent volume of LCBH (see **Figure** **2**a), which shows a huge ≈13% relative volume increase at the endothermic transition corresponding to Δ*V* ≈ 12 · 10^−5^ m^3^ kg^−1^.

High‐pressure differential thermal analysis (HP‐DTA) was carried out in the pressure interval 0 ⩽ *p* ⩽ 0.23 GPa (Figure 2b). At pressures below ≈0.13 GPa, a single peak in the heat flow was measured corresponding to the aforementioned orthorhombic (ordered phase, II) ↔ cubic (disordered phase, I) first‐order phase transition. At pressures above ≈0.13 GPa, the HP‐DTA signals exhibit two peaks thus indicating the appearance of a new phase that we label here as III (high‐pressure enantiotropy). To the best of our knowledge, phase III has not been previously reported in the literature and its specific crystalline structure remains unknown since we did not resolve it. Interestingly, a broad peak was previously detected in differential scanning calorimetry experiments^[^ ^37^ ^]^ that hints at the stabilization of phase III.

Transition temperatures were obtained from the maximum of the HP‐DTA peaks (Figure 2c), thus providing an upper threshold of ≈ (425 K, 0.13 GPa) for the triple point. Considering only the data measured near atmospheric pressure, the pressure dependence of the II→I transition was found to be $\frac{dT}{dp}\approx 420$ K GPa^−1^, which slightly decreases under increasing pressure due to the small convexity of the phase boundary. For the II→III and III→I transitions, linear fits to the obtained phase boundaries yielded $\frac{dT}{dp}\approx 135$ K GPa^−1^ and $\frac{dT}{dp}\approx 310$ K GPa^−1^, respectively. Phase transition entropy changes were calculated via integration of the $\frac{1}{T}\frac{dQ}{dT}$ function after baseline subtraction. As it was already expected, the Δ*S*_II → I_ values associated with the LCBH order–disorder phase transition are noticeably large, namely, ≈208 J K^−1^ kg^−1^ (Figure 2d). By plugging the measured $\frac{dT}{dp}$ and Δ*S*_II → I_ values at atmospheric pressure in the CC equation we obtain Δ*V*_CC_ ≈ 9 · 10^−5^ m^3^ kg^−1^, which is in reasonable agreement with the Δ*V* determined directly from the experiments.

Above *p* ≈ 0.13 GPa, due to the overlapping between the II↔III and III↔I peaks, the contribution associated with each phase transition was decided at the inflection point of the cumulative entropy change function $\int_{T_{1}}^{T}\frac{1}{T^{\prime }}\frac{dQ}{dT^{\prime }}dT^{\prime }$. Δ*S*_t_ remains practically constant from atmospheric pressure all the way up to the triple point. At *p* ≃ 0.13 GPa, we obtained Δ*S*_II → I_ ≈ Δ*S*_II → III_ + Δ*S*_III → I_, as it is required by the condition of thermodynamic equilibrium, while above the triple point Δ*S*_II → III_ ≈ Δ*S*_III → I_. Splitting of the II→I phase transition into II→III and III→I might be associated with the decoupling of the diffusive and orientational degrees of freedom right at the stabilization of the high‐*T* phase, although further investigations are necessary for a conclusive assessment of this hypothesis.

HP‐DTA measurements along with experimental differential scanning calorimetry (Figure S2, Supporting Information), heat capacity (Figure S3, Supporting Information) and theoretical equations of state *V*(*T*, *p*) (i.e., obtained from molecular dynamics simulations, Section 2.3) were used to determine the isobaric entropy curves *S*(*T*, *p*) (Figure S4, Supporting Information), from which BCE can be straightforwardly estimated (Experimental Section). This technique, known as the quasi‐direct method, has been extensively employed in the scientific literature to determine both isothermal entropy and adiabatic temperature changes for a wide variety of caloric materials.^[^ ^5^, ^16^, ^17^, ^18^, ^19^, ^22^, ^23^, ^27^ ^]^ The reliability of the quasi‐direct method has been previously demonstrated through straightforward comparison with direct measurements.^[^ ^46^ ^]^ It is worth noting that in the field of BC effects and materials, direct measurements present challenges due to the typical use of liquids as pressure‐transmitting fluids, which makes achieving adiabatic conditions difficult. This challenge arises from thermal exchange between the barocaloric material and the liquid, resulting in systematically lower reported values than expected.^[^ ^47^, ^48^ ^]^ Alternatively, direct measurements have been reported in systems that eschew any pressure‐transmitting medium, utilizing only a direct piston. However, this approach is viable solely in polymers, as the softness of these materials permits the consideration of uniaxial stress under lateral confinement as nearly hydrostatic.^[^ ^49^, ^50^ ^]^

**Figure 3:**
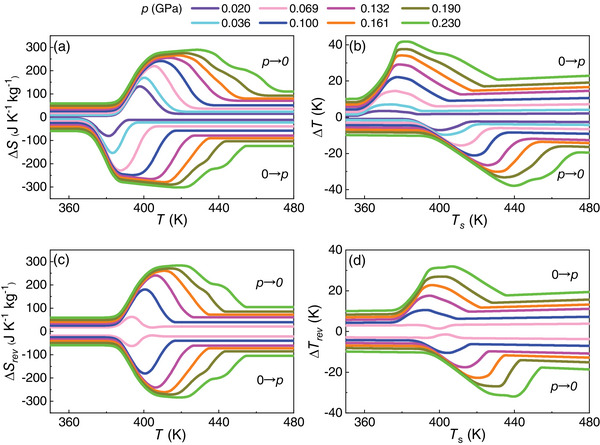
Experimentally measured colossal barocaloric effects in bulk LiCB_11_H_12_. a–c) Isothermal entropy change, Δ*S*, and b–d) adiabatic temperature change, Δ*T*, obtained upon the application and removal of pressure, *p*, considering (a–b) irreversible and (c–d) reversible processes.

**Figures** **3**a,b show representative isothermal entropy changes, |Δ*S*|, and adiabatic temperature changes, |Δ*T*|, obtained upon the first application and removal of the driving pressure shift. It is worth noticing that a small Δ*p* ≈ 0.03 GPa already produced colossal values of |Δ*S*| = 100 J K^−1^ kg^−1^ and |Δ*T*| = 8 K, and similarly Δ*p* ≈ 0.08 GPa yielded |Δ*S*| = 250 J K^−1^ kg^−1^ and |Δ*T*| = 16 K. For the largest pressure shift considered in this study, namely, Δ*p* ≈ 0.23 GPa, the resulting |Δ*S*| and |Δ*T*| amount to 300 J K^−1^ kg^−1^ and 40 K, respectively. We note in passing that the occurrence of two consecutive transitions at pressures above 0.13 GPa serves to broaden the temperature range over which the colossal BC effects manifest in LCBH; however, as depicted in Figure 3, this phenomenon has a minimal impact on the global magnitude of Δ*S* and Δ*T*.

Operation of solid‐state cooling and heating devices requires cyclic application and removal of the driving fields, for which reversible caloric effects, |Δ*S*_rev_| and |Δ*T*_rev_|, must be considered. By reversible caloric effects we mean acquitted of phase transition hysteresis effects.^[^ ^18^ ^]^ The obtained results are shown in Figures 3c,d. Colossal |Δ*S*_rev_| were already obtained for a minimum pressure shift of ≈0.08 GPa. For instance, under a moderate pressure change of ≈0.10 GPa LCBH renders |Δ*S*_rev_| = 200 J K^−1^ kg^−1^ and |Δ*T*_rev_| = 10 K. Meanwhile, for the largest pressure shift considered in this study we measured outstanding values of |Δ*S*_rev_| = 280 J K^−1^ kg^−1^ and |Δ*T*_rev_| = 32 K. In spite of such promising barocaloric features, LCBH is not suitable for domestic cooling or heating applications since the corresponding phase‐transition temperature is above room temperature (Figure 3). Nevertheless, this fact does not thwart the use of this material for thermal management operation since there are many other non‐domestic contexts in which working temperatures range from tens to hundreds of degrees above ambient conditions (e.g., industrial heat pumps and high‐temperature district cooling).

**Figure 4:**
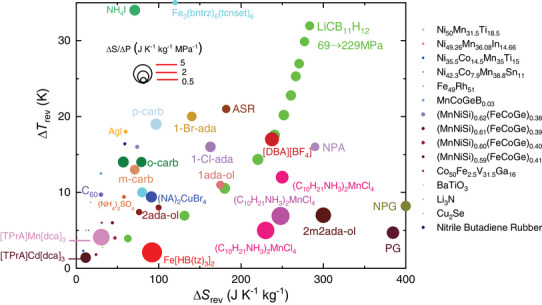
Compendium of experimentally measured reversible BCE. The size of the symbols represents the reversible barocaloric strength defined as the ratio of |Δ*S*_rev_| by the corresponding pressure change Δ*p*. Material names are indicated near each symbol or in the right side of the panel. NPG: neopentylglycol^[^ ^51^ ^]^; PG: pentaglycerine^[^ ^51^ ^]^; NPA: Neopentyl alcohol^[^ ^51^ ^]^; o‐carb: orthocarborane^[^ ^52^ ^]^; m‐carb: metacarborane^[^ ^52^ ^]^; p‐carb: paracarborane^[^ ^52^ ^]^; 1‐Br‐ada: 1‐Bromoadamantane^[^ ^53^ ^]^; 1‐Cl‐ada: 1‐Chloroadamantane^[^ ^53^ ^]^; 1ada‐ol: 1‐adamantanol^[^ ^24^ ^]^; 2ada‐ol: 2‐adamantanol^[^ ^24^ ^]^; 2m2ada‐ol: 2‐methyl‐2‐adamantanol^[^ ^24^ ^]^; ASR: Acetoxy Silicone Rubber^[^ ^54^ ^]^; C_60_^[^ ^55^ ^]^; Fe_3_(bntrz)_6_(tcnset)_6_^[^ ^32^ ^]^; Fe[HB(tz)_3_]_2_^[^ ^56^ ^]^; (C_10_H_21_NH_3_)_2_MnCl_4_^[^ ^23^, ^55^ ^]^; [TPrA]Mn[dca]_3_^[^ ^57^ ^]^; [TPrA]Cd[dca]_3_^[^ ^58^ ^]^; [DBA][BF_4_]^[^ ^25^ ^]^; (NA)_2_CuBr_4_^[^ ^23^ ^]^; NH_4_I^[^ ^59^ ^]^; Nitrile Butadiene Rubber^[^ ^60^ ^]^; Ni_50_Mn_31.5_Ti_18.5_^[^ ^61^ ^]^; Ni_49.26_Mn_36.08_In_14.66_^[^ ^62^ ^]^; Ni_35.5_Co_14.5_Mn_35_Ti_15_^[^ ^63^ ^]^; Ni_42.3_Co_7.9_Mn_38.8_Sn_11.0_^[^ ^64^ ^]^; Fe_49_Rh_51_^[^ ^65^, ^66^ ^]^; MnCoGeB_0.03_^[^ ^67^ ^]^; (MnNiSi)_0.62_(FeCoGe)_0.38_^[^ ^68^ ^]^; (MnNiSi)_0.61_(FeCoGe)_0.39_^[^ ^69^ ^]^; (MnNiSi)_0.60_(FeCoGe)_0.40_^[^ ^69^ ^]^; (MnNiSi)_0.59_(FeCoGe)_0.41_^[^ ^69^ ^]^; Co_50_Fe_2.5_V_31.5_Ga_16_^[^ ^70^ ^]^; BaTiO_3_^[^ ^71^ ^]^; (NH_4_)_2_SO_4_^[^ ^72^ ^]^; AgI^[^ ^73^ ^]^; Li_3_N^[^ ^74^ ^]^; Cu_2_Se.^[^ ^75^ ^]^ Additional details can be found in the Table S1 (Supporting Information).

**Figure** **4** compares most of the experimental |Δ*S*_rev_| and |Δ*T*_rev_| reported thus far in the literature for barocaloric materials. Additionally, the size of the symbols therein accounts for the materials BC strength, which is defined as the ratio of |Δ*S*_rev_| by the corresponding pressure shift Δ*p*. The best performing barocaloric materials, therefore, should appear in the top right side of the panel and with the largest possible symbol area. Each material has been represented with one or two points that best illustrate their overall barocaloric performance, while for LCBH we have selected a set of barocaloric measurements.

Although LCBH is not the best performing material in terms of a single quality, it displays an unprecedentedly well‐balanced and accomplished barocaloric portfolio consisting of colossal |Δ*S*_rev_|, large |Δ*T*_rev_|, and large BC strength obtained under moderate pressure shifts of the order of 0.10 GPa. For instance, in terms of largest |Δ*S*_rev_| the plastic crystal neopentylglycol (NPG) emerges as the clear winner since it holds a gigantic value of ≈400 J K^−1^ kg^−1^^[^ ^18^ ^]^; however, as regards |Δ*T*_rev_| the same material becomes a poor contestant in the presence of LCBH (that is, ≈8 K vs. 32 K). Likewise, the |Δ*T*_rev_| record holder, namely, the spin‐crossover complex Fe_3_(bntrz)_6_(tcnset)_6_,^[^ ^32^ ^]^ presents |Δ*S*_rev_|, and BC strength values that roughly are halves of the LCBH maxima (for instance, ≈120 J K^−1^ kg^−1^ versus 280 J K^−1^ kg^−1^). Therefore, LCBH can be deemed as one of the most thorough and promising barocaloric materials reported to date owing to its unique parity between sizable |Δ*S*_rev_| and |Δ*T*_rev_| obtained under moderate pressure shifts.

### 2.3 Atomistic Simulation of Barocaloric Effects

Hitherto modeling of BCE in plastic crystals has been mostly carried out with the Clausius‐Clapeyron (CC) method.^[^ ^34^, ^36^ ^]^ Nevertheless, the CC method presents several critical drawbacks in the present context: 1) the quantity that is accessed is the phase transition entropy, rather than the barocaloric isothermal entropy change, 2) neither the adiabatic temperature change nor the temperature span over which BCE are operative can be directly estimated, and 3) partial entropy contributions stemming from different degrees of freedom (i.e., vibrational, molecular orientational and ion diffusive) cannot be determined. These technical limitations preclude any quantitative and physically meaningful comparison with the experiments. Here, we use a simulation approach previously developed by some of us that is able to overcome such shortcomings and provide a fair theoretical description of BCE in LCBH (Experimental Section).^[^ ^76^ ^]^

**Figure 5:**
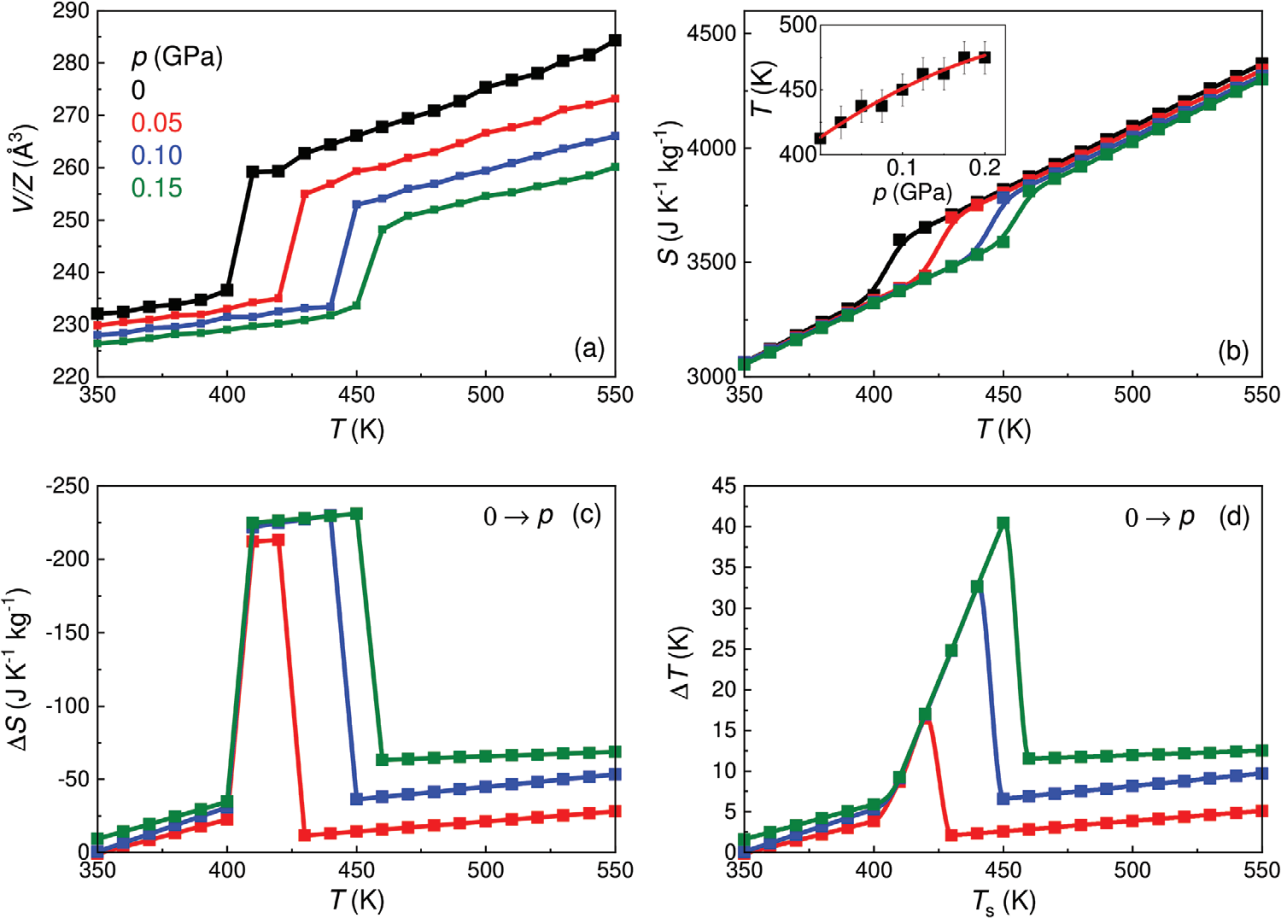
Colossal BCE estimated for bulk LiCB_11_H_12_ with MD simulations. a) Volume change per formula unit across the phase transition expressed as a function of temperature and pressure. b) Total entropy curves expressed as a function of pressure and temperature. Inset: theoretically calculated *p*–*T* phase diagram. c) Isothermal entropy and d) adiabatic temperature changes expressed as a function of temperature and pressure. Results were obtained from *NpT*‐MD simulations.

**Figure 6:**
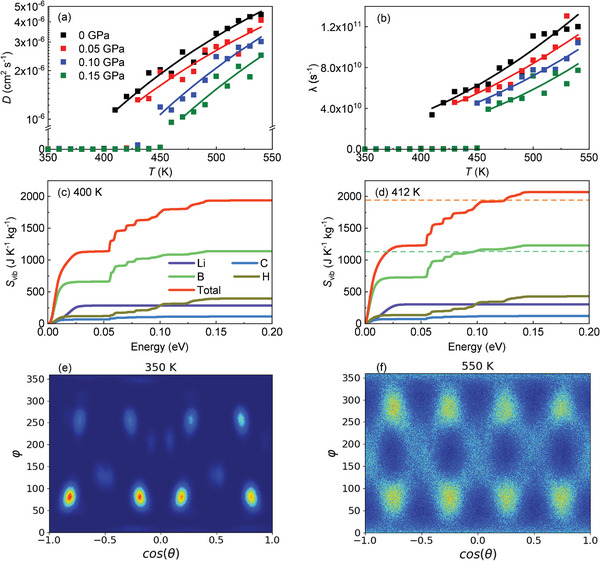
Atomistic insights into the order‐disorder phase transition in LiCB_11_H_12_ from MD simulations. a) Lithium ion diffusion coefficient, *D*_Li_. b) Anionic reorientational frequency, λ_CBH_. Solid lines correspond to Arrhenius law fits. c–d) Cumulative function of the vibrational entropy as a function of the phonon energy and atomic species, calculated for the ordered (*T* = 400 K) and disordered (*T* = 412 K) phases at zero pressure. Dashed lines indicate analogous asymptotic values reached in the ordered phase. e–f) Angular probability density function estimated for the molecular (CB_11_H_12_)^−^ anions calculated in the ordered (*T* = 350 K) and disordered (*T* = 550 K) phases at zero pressure, expressed as a function of the polar (θ) and azimuthal (ϕ) angles. Dark and bright areas represent low and high probability regions, respectively.

**Figures** **5**a,b show the theoretical equation of state *V*(*T*, *p*) and *p*–*T* phase diagram of bulk LCBH obtained from molecular dynamics (MD) simulations (Experimental Section). We determined the phase boundary of the high‐*T* (disordered) and low‐*T* (ordered) phases by conducting numerous MD simulations at small *p*–*T* shifts of 0.025 GPa and 12.5 K. Each phase boundary point in Figure 5b (inset) corresponds to sharp and simultaneous changes in the volume, Li^+^ diffusion coefficient (*D*_Li_), and molecular [CB_11_H_12_]^−^ orientational frequency (λ_CBH_), as identified in the MD simulations (**Figures** **6**a,b). At zero pressure, we estimated a huge volume increase of about 11% at the theoretical transition temperature *T*_*t*_ ≈ 400 K, along with the order parameter changes Δ*D*_Li_ = 1.13 · 10^−6^ cm^2^ s^−1^ and Δλ_CBH_ = 0.33 · 10^11^ s^−1^. It was found that the pressure dependence of the transition temperature could be precisely reproduced by the second‐order polynomial curve *T*_*t*_(*p*) = 412 + 438*p* − 610*p* ^2^ (red line in the inset of Figure 5b), in which the temperature and pressure are expressed in units of K and GPa, respectively. The slight $\frac{dT}{dp}$ decrease under increasing compression is consistent with the *p*‐induced reduction of the transition volume change since Δ*S*_*t*_ is roughly independent of pressure, in agreement with our experiments. It is worth noting that the experimentally observed phase‐transition hysteresis cannot be reliably reproduced by the equilibrium MD approach employed in this study.^[^ ^36^ ^]^ In particular, the low symmetry of the CB_11_H_12_ anions in LCBH prevents the restoration of a fully ordered state upon cooling within a feasible time scale (≈ ns) in our simulations.

The LCBH *p*–*T* phase diagram obtained from MD simulations (Figure 5b) is in quantitative good agreement with the experiments performed below the triple point at *p* ≈ 0.13 GPa (Figure 2b), although the transition temperatures are slightly overestimated by theory. For example, at zero pressure and *p* = 0.10 GPa the MD simulations yielded *T*_*t*_ = 410 ± 15 and 440 ± 15 K (Figure 5), respectively, to be compared with the corresponding experimental values 390 ± 10 and 410 ± 10 K (Figure 2b). The agreement between the predicted and measured volumes for the ordered and disordered phases at zero pressure is also notable, finding only small relative discrepancies of ≈ 1% for the low‐*T* phase (Figures 2a and 5a). Meanwhile, the triple point observed in the experiments was not reproduced by the MD simulations. It is worth noting, however, that under *p* ≠ 0 conditions and close to *T*_*t*_ we observed pre‐transitional effects in our simulations consisting of few slowly diffusing Li ions in the ordered phase (Figure S5, Supporting Information).

Figure 5c,d show the theoretical barocaloric |Δ*S*| and |Δ*T*| deduced from the entropy curves *S*(*p*, *T*) enclosed in Figure 5b, which were obtained from data generated in the MD simulations. The agreement between these theoretical results and the corresponding experimental values is remarkably good for pressures below the experimental triple point. For example, for a pressure shift of 0.10 GPa, we estimated an isothermal entropy change of 227 J K^−1^ kg^−1^ and an adiabatic temperature change of 32 K from the MD simulations, to be compared with the corresponding experimental values 250 J K^−1^ kg^−1^ and 24 K (Figure 3a,b). In view of such a notable agreement, we characterized with MD simulations the contributions to the phase transition entropy change stemming from the vibrational, molecular orientational, and cation diffusive degrees of freedom, a highly valuable atomistic insight that in principle cannot be obtained from the experiments.

Figure 6a,b reveal synchronized surges in *D*_Li_ and λ_CBH_ at the order‐disorder phase transition points. Thus, both ion diffusion and molecular anion orientational disorder (Figure 6e,f) contribute to the transition entropy change and barocaloric effects disclosed in LCBH. Nevertheless, there is a third possible source of entropy in the crystal which is related to the lattice vibrations, *S*_vib_ (Figures S6–S7, Supporting Information). Figure 6c,d show examples of the cumulative *S*_vib_ function expressed as a function of the vibrational phonon energy, calculated for LCBH in the ordered and disordered phases at zero pressure and evaluated for each atomic species. Therein, it is appreciated that the largest contribution to the *S*_vib_ difference between the order and disordered phases comes from the B atoms (followed by hydrogen). This outcome can be rationalized in terms of the relative great abundance of this species in LCBH (≈45%) and its larger mass as compared to that of H atoms (ten times heavier): B ions have a predominant weight on the low‐frequency vibrational modes (Figure 6c,d) that most significantly contribute to *S*_vib_ near ambient temperature.

**Figure 7:**
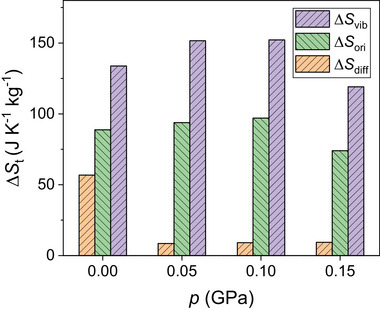
Partial contributions to the entropy change accompanying the order‐disorder phase transition in LCBH expressed as a function of pressure. Entropy changes stem from the vibrational, Δ*S*_vib_, molecular orientational, Δ*S*_ori_, and cation diffusive, Δ*S*_diff_, degrees of freedom. Results were obtained from comprehensive molecular dynamics simulations and Gibbs free energy calculations (Experimental Section).

**Figure** **7** shows the relative contributions of the vibrational, molecular orientational, and ion diffusion degrees of freedom to the phase transition entropy change estimated at different pressures with MD simulations. Interestingly, in all the analyzed cases the largest contribution stems from changes in the lattice vibrations, Δ*S*_vib_, followed by the molecular reorientations, Δ*S*_ori_, and finally ion diffusion, Δ*S*_diff_. For example, at zero pressure the vibrational, molecular orientational and ion diffusive degrees of freedom respectively contribute in ≈48, 32, and 20% to Δ*S*_*t*_. The entropy preeminence of the lattice vibrations can be rationalized in terms of (1) the huge volume expansion accompanying the order‐disorder phase transition (≈10%, Figure 5a), which further curtails the frequency of the low‐energy phonon bands in the disordered phase (Figure S6, Supporting Information), and (2) the intensification and amplitude broadening of the molecular libration modes in the disordered phase (inferred from the angular probability density variations around the equilibrium positions in Figure 6e,f).

These outcomes are highly valuable and physically insightful since thus far molecular reorientations were thought to be the primary source of entropy variation in plastic crystals undergoing order‐disorder phase transitions.^[^ ^33^, ^34^, ^35^ ^]^ Moreover, our theoretical findings appear to be consistent with recent experiments performed for the archetypal plastic crystal adamantane^[^ ^77^ ^]^ and the orientationally disordered ferroelectric ammonium sulfate,^[^ ^78^ ^]^ which show that the vibrational contributions to the order–disorder phase entropy change surpass those resulting from molecular reorientations.

The vibrational and orientational entropy changes remain more or less constant for pressures ⩽0.1 GPa, whereas Δ*S*_diff_ significantly decreases under compression. For instance, at 0.1 GPa the diffusive degrees of freedom contribute to Δ*S*_*t*_ in less than 4%. These outcomes can be understood in terms of the small fraction of diffusive ions in LCBH (i.e., one Li atom per formula unit) and the marked decline in *D*_Li_ induced by pressure (Figure 6a). The appearance of pre‐transitional effects in our MD simulations, specially under *p* ≠ 0 conditions (Figure S5, Supporting Information), also contributes to the noticeable Δ*S*_diff_ drop caused by compression. Nonetheless, it is worth noting that despite the relative minuteness of Δ*S*_diff_, cation disorder was found to play a critical role on triggering molecular orientational disorder, which by contrast contributes very significantly to Δ*S*_*t*_. In particular, we conducted constrained MD runs in which we fixed the positions of the lithium ions so that they could not diffuse. It was found then that molecular orientational disorder only emerged at temperatures well above 550 K (Figure S8, Supporting Information). (Conversely, we fixed the positions of the molecular anions and left the lithium cations to freely evolve, finding that ionic transport was significantly low; thus, it appears that the conventional “paddle‐wheel” mechanism,^[^ ^79^ ^]^ in which anion rotational dynamics constructively contributes to ionic diffusion, is relevant for LCBH.^[^ ^45^ ^]^) It can be concluded, therefore, that cation disorder crucially assists on the realization of colossal BCE through the order–disorder phase transition, a characteristic trait that differentiates LCBH from other molecular plastic crystals bearing also great barocaloric promise.

## 3 Conclusion

Colossal barocaloric effects (BCE) driven by pressure shifts of the order of 0.10 GPa were experimentally and theoretically disclosed in bulk LiCB_11_H_12_ (LCBH), a compound that at high temperatures presents disorder features characteristic of both plastic crystals and superionic materials, namely, molecular reorientational motion and ion diffusion. Reversible peaks of |Δ*S*_rev_| = 280 J K^−1^ kg^−1^ and |Δ*T*_rev_| = 32 K were experimentally measured around 400 K for a pressure shift of 0.23 GPa, yielding huge and reversible barocaloric strengths of ≈2 J K^−1^ kg^−1^ MPa^−1^ over an ample temperature interval of several tens of kelvin. Likewise, for a smaller pressure shift of 0.10 GPa, we obtained very promising values of |Δ*S*_rev_| = 200 J K^−1^ kg^−1^ and |Δ*T*_rev_| = 10 K. These results place LCBH among the best‐known barocaloric materials in terms of huge and reversible isothermal entropy and adiabatic temperature changes, two quantities that rarely are found simultaneously in a same material.

Atomistic molecular dynamics simulations yielded theoretical |Δ*S*| and |Δ*T*| in very good agreement with the experimental values, and allowed to quantify the importance of vibrational, molecular orientational, and ion diffusive degrees of freedom on the disclosed colossal BCE. It was found that the contribution to the phase transition entropy change stemming from the lattice vibrations was the largest, followed by that of molecular reorientations and both being much superior than the entropy associated with lithium diffusion alone. Nevertheless, cationic disorder was found to have a critical influence on the stabilization of orientational disorder thus, in spite of its small contribution to Δ*S*_*t*_, lithium diffusion appears to be essential for the emergence of colossal BCE in bulk LCBH. These results are of high significance since reveal the preeminence of the vibrational degrees of freedom in the phase transition entropy change of a plastic crystal, and demonstrate atomistic BCE mechanisms other than molecular reorientational disorder (i.e., lattice vibrations and ion diffusion).

LCBH belongs to the family of closo‐borate materials, a promising class of solid electrolytes for all‐solid‐state batteries. Examples of akin compounds that have already been synthesized in the laboratory and tested for electrochemical energy storage applications are NaCB_11_H_12_,^[^ ^37^, ^40^ ^]^ KCB_11_H_12_,^[^ ^42^ ^]^ and LiCB_9_H_10_.^[^ ^43^, ^44^ ^]^ Colossal BCE induced by hydrostatic pressure changes could also exist in these materials and in other similar compounds harboring both ion diffusion and molecular orientational disorder at or near room temperature. Thus, the present combined experimental‐theoretical study opens new horizons in solid‐state cooling and heating and advances knowledge in the realization of colossal BCE in plastic crystals.

## 4 Experimental Section

### Experimental Techniques

*Materials synthesis* LiCB_11_H_12_ was obtained by drying the hydrated compound LiCB_11_H_12_·xH_2_O (Katchem, Ltd.) under vacuum (<5 × 10^−4^ Pa) at 160 °C for 12 h.

*X‐ray powder diffraction* High‐resolution X‐ray powder diffraction measurements were performed using the Debye–Scherrer geometry and transmission mode with a horizontally mounted cylindrical position‐sensitive INEL detector (CPS‐120). Monochromatic Cu‐Kα_1_ radiation was selected by means of a curved germanium monochromator. Temperature‐dependent measurements were performed using a liquid nitrogen 700 series Oxford Cryostream Cooler. Powder samples were introduced into 0.5 mm diameter Lindemann capillaries. Volume was obtained by pattern matching procedure. Regarding the crystallographic model fitted to the experimental data, the lattice parameters were determined using pattern matching via the Le Bail fitting integrated into the FullProf software. For the low‐temperature phase, the lattice and space group proposed by Tang *et al.* were utilized.^[^ ^37^ ^]^ For the high‐temperature phase, the observed Bragg reflections suggested an fcc cubic phase with parameters similar to those published by Tang et al.^[^ ^37^ ^]^ No attempt was made to confirm the possible existence of new polymorphs in LCBH at temperatures exceeding 400 K.^[^ ^37^ ^]^

*Quasi‐direct barocaloric measurements* A Q100 thermal analyzer (TA Instruments) was used to perform differential scanning calorimetry experiments at atmospheric pressure with ≈ 10 mg of sample hermetically encapsulated in Aluminum pans (Figure S2, Supporting Information). The standard mode (at 3, 5, and 10 K min^−1^) was used to determine the transition properties whereas the modulated mode (isothermal conditions, modulation amplitude 1 °C, modulation period 120 s) was used to measure the heat capacity in each phase (Figure S3, Supporting Information).

Pressure‐dependent calorimetry was performed with a custom‐built high‐pressure differential thermal analyzer (from Irimo, Bellota Herramientas S.A.) that uses Bridgman thermocouples as thermal sensors. The nominal operational pressure range was from atmospheric to 0.3 GPa and the temperature range from room temperature to 473 K. Heating ramps were performed at 3 K min^−1^ using a resistive heater whereas cooling were carried out at ≈−2 K min^−1^ by an air stream. A few hundreds of mg of LiCB_11_H_12_ were mixed with an inert perfluorinated fluid (Galden Bioblock Scientist), to remove air, and sealed within tin capsules. The pressure‐transmitting fluid was Therm240 (Lauda).

Isobaric entropy functions *S*(*T*, *p*) were determined with respect to a reference temperature *T*_0_ below the transition using the method explained in ref. [55] (Figure S4, Supporting Information). The procedure is based on the following thermodynamic equation:

(1) $$S\left(T,p\right)=S\left(T_{0},p\right)+\int_{T_{0}}^{T}\frac{1}{T}\left(C_{p}+\frac{dQ}{dT}\right)dT\;$$

where $\frac{dQ}{dT}$ is the heat flow in temperature due to the first‐order phase transition measured by pressure‐dependent calorimetry.

In each phase, *C*_*p*_ is the corresponding heat capacity and was considered independent of pressure as indicated by the approximately linear behavior of volume with temperature obtained in the two phases from MD simulations (Figure 5) along with the thermodynamic equation:

(2) $${\left(\frac{\partial C_{p}}{\partial p}\right)}_{T}=-T{\left(\frac{\partial^{2}V}{\partial T^{2}}\right)}_{p}\;$$

In the transition region *C*_*p*_ was calculated as an average weighted according to the fraction of each phase. To take into account the dependence of the transition region with pressure, the overall *C*_*p*_ function at atmospheric pressure obtained in each phase and across the transition was extrapolated to higher temperatures according to the experimental value of $\frac{dT}{dp}\Delta p$, where Δ*p* is the pressure change applied in each particular case. Experimental measurement of *C*_*p*_ at atmospheric pressure and the calculated curves at different pressures are shown in Figure S3 (Supporting Information).

The pressure dependence of *S*(*T*, *p*) was evaluated using the thermodynamic equation:

(3) $$S\left(T,p\right)=S\left(T,p_{0}\right)-\int_{p_{0}}^{p}{\left(\frac{\partial V}{\partial T}\right)}_{T,p^{\prime }}dp^{\prime }\;$$

where *p*_0_ was selected equal to *p*_atm_ = 1 bar. Here, the approximation ${\left(\frac{\partial V}{\partial T}\right)}_{T,p}≃{\left(\frac{\partial V}{\partial T}\right)}_{T,p_{0}}$ was employed, which was reasonable based on the ${\left(\frac{\partial V}{\partial T}\right)}_{T,p}$ data obtained from the MD simulations (Figure 5).

Once the entropy function *S*(*T*, *p*) was determined for both heating and cooling runs independently (Figure S4, Supporting Information), BCE obtained upon first application or removal of the field were calculated as:

(4) $$\begin{array}{c} \Delta S\left(T,p_{0}\to p_{1}\right)=S\left(T,p_{1}\right)-S\left(T,p_{0}\right)\;\mathrm{and} \end{array}$$

(5) $$\begin{array}{c} \Delta T\left(T_{s},p_{0}\to p_{1}\right)=T\left(S,p_{1}\right)-T_{s}\left(T,p_{0}\right)\; \end{array}$$

where *T*_*s*_ is the starting temperature of the heating/cooling process. Here, it must be considered that for materials with $\frac{dT}{dp}>0$ BCE on compression (*p*_0_ = *p*_atm_, *p*_1_ > *p*_atm_) and decompression (*p*_0_ > *p*_atm_, *p*_1_ = *p*_atm_) are calculated from *S*(*T*, *p*) functions obtained on cooling and heating, respectively.^[^ ^18^ ^]^ Meanwhile, BCE obtained reversibly on cyclic compression–decompression processes were calculated from the *S*(*T*, *p*) curves obtained on heating at atmospheric pressure and cooling at high pressure.

### Simulation Techniques

*—Molecular dynamics simulations* Force‐field based molecular dynamics (MD) simulations were performed using a previously reported interatomic potential for LiCB_11_H_12_.^[^ ^45^ ^]^ This force field was a combination of Coulomb‐Buckingham (CB), harmonic bond, and angle‐type potentials, namely:

(6a) $$\begin{array}{c} U\left(r,\theta \right)=U_{\mathrm{CB}}\left(r\right)+U_{\mathrm{bond}}\left(r\right)+U_{\mathrm{angle}}\left(\theta \right)\; \end{array}$$

(6b) $$\begin{array}{c} U_{\mathrm{CB}}\left(r\right)=\frac{q_{i}q_{j}}{4\pi \varepsilon_{0}r}+A_{ij}\exp\left(-r/\rho \right)-\frac{C_{ij}}{r^{6}}\; \end{array}$$

(6c) $$\begin{array}{c} U_{\mathrm{bond}}\left(r\right)=\frac{1}{2}k_{r}{\left(r-r_{0}\right)}^{2}\;\mathrm{and} \end{array}$$

(6d) $$\begin{array}{c} U_{\mathrm{angle}}\left(\theta \right)=\frac{1}{2}k_{\theta }{\left(\theta -\theta_{0}\right)}^{2}\; \end{array}$$

where *q*_*i*_ denotes the charge of the ion labeled *i*, ϵ_0_ the vacuum permittivity, *A*_*ij*_ and ρ the short‐range repulsive energy and length scales for the pairs of atoms *ij*, and *C*_*ij*_ the corresponding dispersion interaction coefficient. *r*_0_ and θ_0_ are an equilibrium bond distance and angle, respectively, and *k*_*r*_ and *k*_θ_ the spring constants of the harmonic bond and angle potentials. The numerical value of these potential parameters can be found in the Table S2 (Supporting Information).

*NpT*‐MD simulations were performed in the temperature range 325 ⩽ *T* ⩽ 525 K at intervals of 12.5 K, and pressure range 0 ⩽ *p* ⩽ 0.15 GPa at intervals of 0.025 GPa. The temperature and pressure in the system were controlled with thermostating and barostating techniques, in which some dynamic variables were coupled with the particle velocities and simulation box dimensions. The simulation supercell comprised a total of 6400 atoms. The simulations were initialized considering a single phase (i.e., phase coexistence simulations were not performed). A time step of 0.5 fs was employed for integration of the atomic forces along with the velocity Verlet algorithm. A typical *NpT*‐MD run lasted for about 2 ns and the atomic trajectories were stored at intervals of 500 fs. Detailed analyses and statistical time averages were performed over the last 1 ns of such simulations. To ensure proper convergence of the estimated thermodynamic properties, the convergence of the vibrational entropy was investigated as a function of simulation length (Figure S9, Supporting Information). Additionally, in a few instances, the thermalization runs were validated by employing much longer thermalization simulation times of 10 ns. Periodic boundary conditions were applied along the three Cartesian directions and the Ewald summation technique was used for evaluation of the long‐range Coulomb interactions with a short‐range cut‐off distance of 13 Å. All the *NpT*‐MD simulations were carried out with the LAMMPS software package.^[^ ^80^ ^]^

*Density functional theory and ab initio molecular dynamics simulations* First‐principles calculations based on density functional theory (DFT) were performed to analyze the energy, structural and vibrational properties of bulk LCBH. The DFT calculations were carried out with the VASP code^[^ ^81^ ^]^ by following the generalized gradient approximation to the exchange‐correlation energy due to Perdew et al. (PBE).^[^ ^82^ ^]^ The projector augmented‐wave method was used to represent the ionic cores,^[^ ^83^ ^]^ and the electronic states 1*s*–2*s* Li, 2*s*–2*p* C, 2*s*–2*p* B and 1*s* H were considered as valence. Wave functions were represented in a plane‐wave basis truncated at 650 eV. By using these parameters and dense k‐point grids for Brillouin zone integration, the resulting energies were converged to within 1 meV per formula unit. In the geometry relaxations, a tolerance of 0.005 eV Å^−−1^ was imposed in the atomic forces.

Ab initio molecular dynamics (AIMD) simulations based on DFT were carried out to assess the reliability of the interatomic potential model employed in the MD simulations on the description of the vibrational degrees of freedom of bulk LCBH (Figure S7, Supporting Information). The AIMD simulations were performed in the canonical ensemble (*N*, *V*, *T*) considering constant number of particles, volume, and temperature. The constrained volumes were equal to the equilibrium volumes determined at zero temperature, an approximation that had been shown to be reasonable at moderate temperatures.^[^ ^84^ ^]^ The temperature in the AIMD simulations was kept fluctuating around a set‐point value by using Nose‐Hoover thermostats. A large simulation box containing 800 atoms was employed in all the simulations, and periodic boundary conditions were applied along the three Cartesian directions. Newton's equations of motion were integrated by using the customary Verlet's algorithm and a time‐step length of δ*t* = 10^−3^ ps. Γ‐point sampling for integration within the first Brillouin zone was employed in all the AIMD simulations. The AIMD simulations comprised long simulation times of ≈200 ps and temperatures in the range 200 ⩽ *T* ⩽ 500 K.

*Estimation of key quantities with MD simulations* The mean square displacement of the lithium ions was estimated with the formula^[^ ^84^ ^]^:

(7) $$\begin{array}{ccc} {\mathrm{MSD}}_{\mathrm{Li}}\left(\tau \right) & = & \frac{1}{N_{\mathrm{ion}}\left(N_{\mathrm{step}}-n_{\tau }\right)}\\ & & \times \sum_{i=1}^{N_{\mathrm{ion}}}\sum_{j=1}^{N_{\mathrm{step}}-n_{\tau }}{\left|r_{i}\left(t_{j}+\tau \right)-r_{i}\left(t_{j}\right)\right|}^{2}\; \end{array}$$

where **r**_*i*_(*t*_*j*_) is the position of the migrating ion *i* at time *t*_*j*_ (=*j* · δ*t*), τ represents a lag time, *n*_τ_ = τ/δ*t*, *N*_ion_ is the total number of mobile ions, and *N*_step_ the total number of time steps. The maximum *n*_τ_ was chosen equal to *N*_step_/2, hence sufficient statistics were accumulated to reduce significantly the fluctuations in ${\mathrm{MSD}}_{\mathrm{Li}}\left(\tau \right)$ at large τ's. The diffusion coefficient of lithium ions was calculated with the Einstein's relation:

(8) $$D_{\mathrm{Li}}=\lim_{\tau \to \infty }\frac{{\mathrm{MSD}}_{\mathrm{Li}}\left(\tau \right)}{6\tau }\;$$

by performing linear fits to the averaged MSD_Li_ values calculated at long τ.

The angular autocorrelation function of the molecular [CB_11_H_12_]^−^ anions was estimated using the expression^[^ ^36^ ^]^:

(9) $$\phi_{\mathrm{CBH}}\left(\tau \right)=\left\langle \hat{r}\left(t\right)\cdot \hat{r}\left(t+\tau \right)\right\rangle \;$$

where $\hat{r}$ is a unitary vector connecting the center of mass of each closoborane unit with one of its edges and 〈⋅⋅⋅〉 denotes statistical average in the (*N*, *p*, *T*) ensemble considering all the molecular anions. This autocorrelation function typically decays as ∝exp [− λ_CBH_ · τ], where the parameter λ_CBH_ represents a characteristic reorientational frequency. For significant anion reorientational motion, that is, large λ_CBH_, the ϕ_CBH_ function decreases rapidly to zero with time.

The temperature dependence of the lithium diffusion coefficient was assumed to follow an Arrhenius law at any pressure of the form:

(10) $$D_{\mathrm{Li}}\left(T\right)=D_{0}\cdot e^{-(\frac{E_{a}}{k_{B}T})}\;$$

where *D*_0_ and *E*_*a*_ are parameters that depend on *p* and *k*_*B*_ represents the Boltzmann constant. The reorientational frequency of closoborane units, λ_CBH_, was assumed to follow a similar dependence on temperature.

The entropy of each phase was calculated as a function of temperature and pressure, *S*(*p*, *T*), by fully considering the vibrational, molecular orientational and ion diffusive degrees of freedom^[^ ^76^ ^]^:

(11) $$S\left(p,T\right)=S_{\mathrm{vib}}\left(p,T\right)+S_{\mathrm{ori}}\left(p,T\right)+S_{\mathrm{diff}}\left(p,T\right)\;$$

In the low‐*T* phase, *S*_ori_ and *S*_diff_ are null while in the high‐*T* phase are finite and positive.

The vibrational density of states (VDOS), *g*(ω), was calculated via the Fourier transform of the velocity–velocity autocorrelation function obtained directly from the *NpT*‐MD simulations, namely:

(12) $$g\left(\omega \right)=\frac{1}{N_{ion}}\sum_{i}^{N_{ion}}\int_{0}^{\infty }\left\langle v_{i}\left(\tau \right)\cdot v_{i}\left(0\right)\right\rangle e^{i\omega \tau }d\tau \;$$

where **v**_*i*_(*t*) represents the velocity of the atom labeled *i* at time *t*, and 〈⋅⋅⋅〉 denotes statistical average in the (*N*, *p*, *T*) ensemble. The vibrational entropy was subsequently estimated with the formula^[^ ^76^, ^85^ ^]^:

(13) $$\begin{array}{ccc} S_{\mathrm{vib}}\left(p,T\right) & = & -\int_{0}^{\infty }k_{B}\ln\left[2\sinh\left(\frac{\hbar \omega }{2k_{B}T}\right)\right]\hat{g}\left(\omega \right)d\omega \\ & & +\int_{0}^{\infty }\frac{\hbar \omega }{2T}\tanh^{-1}\left(\frac{\hbar \omega }{2k_{B}T}\right)\hat{g}\left(\omega \right)d\omega \; \end{array}$$

where $\hat{g}\left(\omega \right)$ is the normalized vibrational density of states ($\int_{0}^{\infty }\hat{g}\left(\omega \right)d\omega =3N_{ion}$) and the dependence on pressure (and also temperature) is implicitly contained in $\hat{g}\left(\omega \right)$.

The orientational entropy of the molecular anions, *S*_ori_, was directly calculated from the angular probability density, ρ(θ, ϕ), like Refs. [76, 86]:

(14) $$S_{\mathrm{ori}}\left(p,T\right)=-k_{B}\int_{0}^{\pi }\int_{0}^{2\pi }\rho \left(\theta ,\phi \right)\ln\rho (\theta ,\phi )\;d\cos\theta d\phi \;$$

where θ and ϕ are the polar and azimuthal angles as referred to a fixed arbitrary axis in each molecule, and ρ(θ, ϕ) was obtained from the *NpT*‐MD simulation runs in the form of average histograms (Figure 6). The orientational entropy was offset by a same amount in the low‐*T* ordered and high‐*T* disordered phases in order to reproduce the condition *S*_ori_ = 0 at *T* ⩽ *T*_*t*_.

The ion diffusive entropy difference was estimated at the phase transition points via equalization of the Gibbs free energies of the low‐*T* (O) and high‐*T* (D) phases, namely, *G* ^*D*^(*p*, *T*_*t*_) = $G^{O}$(*p*, *T*_*t*_), thus leading to the expression:

(15) $$\Delta S_{\mathrm{diff}}\left(p,T_{t}\right)=\frac{\left\langle \Delta E\right\rangle }{T_{t}}+p\frac{\left\langle \Delta V\right\rangle }{T_{t}}-\Delta S_{\mathrm{vib}}-\Delta S_{\mathrm{ori}}\;$$

where Δ*X* ≡ *X* ^*D*^ − $X^{O}$ and *E* represents the internal energy of the system. For any pressure, Δ*S*_diff_ was assumed to be constant at temperatures *T*_*t*_ ⩽ *T*.

## Conflict of Interest

The authors declare no conflict of interest.

## Supporting information

Supporting Information

## Data Availability

The data that support the findings of this study are available from the corresponding author upon reasonable request.

## References

[1] X. Moya , N. D. Mathur , Science 2020, 370, 797. 33184207 10.1126/science.abb0973

[2] R. Bergamini , J. K. Jensen , B. Elmegaard , Energy 2019, 182, 110.

[3] M. Qu , O. Abdelaziz , H. Yin , Energy Convers. Manage. 2014, 87, 175.

[4] N. Fernandez , Y. Hwang , R. Radermacher , Int. J. Refrig. 2010, 33, 635.

[5] L. Mañosa , A. Planes , M. Acet , J. Mater. Chem. A 2013, 1, 4925.

[6] H. Hou , S. Qian , I. Takeuchi , Nat. Rev. Mater. 2022, 7, 633.

[7] J. García‐Ben , J. López‐Beceiro , R. Artiaga , J. Salgado‐Beceiro , I. Delgado‐Ferreiro , Y. V. Kolen'ko , S. Castro‐García , M. A. Señarís‐Rodríguez , M. Sánchez‐Andújar , J. M. Bermúdez‐García , Chem. Mater. 2022, 34, 3323. 35444364 10.1021/acs.chemmater.2c00137 PMC9011131

[8] J. María Gelpi , García‐Ben, J. López‐Beceiro , R. Artiaga , J. Salgado‐Beceiro , I. Delgado‐Ferreiro , Y. V. Kolen'ko , S. Castro‐García , M. A. Señarís‐Rodríguez , M. Sánchez‐Andújar , J. M. Bermúdez‐García , Adv. Mater. 2024, 36, 2310499.

[9] Y. Wang , Z. Zhang , T. Usui , M. Benedict , S. Hirose , J. Lee , J. Kalb , D. Schwartz , Science 2020, 370, 129. 33004523 10.1126/science.aba2648

[10] I. Takeuchi , K. Sandeman , Phys. Today 2015, 68, 48.

[11] (Ed.: P. Lloveras ), Barocaloric Effects in the Solid State, IOP Publishing, Bristol, England 2023, pp. 2053–2563.

[12] L. Mañosa , D. González‐Alonso , A. Planes , E. Bonnot , M. Barrio , J.‐L. Tamarit , S. Aksoy , M. Acet , Nat. Mater. 2010, 9, 478. 20364140 10.1038/nmat2731

[13] L. Mañosa , A. Planes , Adv. Mater. 2017, 29, 1603607. 10.1002/adma.201603607 28026063

[14] C. Cazorla , Appl. Phys. Rev. 2019, 6, 041316.

[15] P. Lloveras , J.‐L. Tamarit , MRS Energy Sustain. 2021, 8, 3.

[16] P. Lloveras , A. Aznar , M. Barrio , P. Negrier , C. Popescu , A. Planes , L. Mañosa , E. Stern‐Taulats , A. Avramenko , N. D. Mathur , X. Moya , J.‐L. Tamarit , Nat. Commun. 2019, 10, 1803. 31000715 10.1038/s41467-019-09730-9 PMC6472423

[17] B. Li , Y. Kawakita , S. Ohira‐Kawamura , T. Sugahara , H. Wang , J. Wang , Y. Chen , S. I. Kawaguchi , S. Kawaguchi , K. Ohara , K. Li , D. Yu , R. Mole , T. Hattori , T. Kikuchi , S.‐i. Yano , Z. Zhang , Z. Zhang , W. Ren , S. Lin , O. Sakata , K. Nakajima , Z. Zhang , Nature 2019, 567, 506. 30918372 10.1038/s41586-019-1042-5

[18] A. Aznar , P. Lloveras , M. Barrio , P. Negrier , A. Planes , L. Mañosa , N. D. Mathur , X. Moya , J.‐L. Tamarit , J. Mater. Chem. A 2020, 8, 639.

[19] A. Aznar , P. Negrier , A. Planes , L. Mañosa , E. Stern‐Taulats , X. Moya , M. Barrio , J.‐L. Tamarit , P. Lloveras , Appl. Mater. Today 2021, 23, 101023.

[20] K. Zhang , R. Song , J. Qi , Z. Zhang , Z. Zhang , C. Yu , K. Li , Z. Zhang , B. Li , Adv. Funct. Mater. 2022, 32, 2112622.

[21] W. Imamura , É. O. Usuda , L. S. Paixão , N. M. Bom , A. M. Gomes , A. M. G. Carvalho , Chin. J. Polym. Sci. 2020, 38, 999.

[22] J. Li , M. Barrio , D. J. Dunstan , R. Dixey , X. Lou , J.‐L. Tamarit , A. E. Phillips , P. Lloveras , Adv. Funct. Mater. 2021, 31, 2105154.

[23] J. Seo , R. D. McGillicuddy , A. H. Slavney , S. Zhang , R. Ukani , A. A. Yakovenko , S.‐L. Zheng , J. A. Mason , Nat. Commun. 2022, 13, 2536. 35534457 10.1038/s41467-022-29800-9 PMC9085852

[24] A. Salvatori , P. Negrier , A. Aznar , M. Barrio , J. L. Tamarit , P. Lloveras , APL Mater. 2022, 10, 111117.

[25] J. García‐Ben , J. M. Bermúdez‐García , R. J. C. Dixey , I. Delgado‐Ferreiro , A. L. Llamas‐Saiz , J. López‐Beceiro , R. Artiaga , A. García‐Fernández , U. B. Cappel , B. Alonso , S. Castro‐García , A. E. Phillips , M. Sánchez‐Andújar , M. A. Señarís‐Rodríguez , J. Mater. Chem. A 2023, 11, 22232.

[26] J. Seo , R. Ukani , J. Zheng , J. D. Braun , S. Wang , F. E. Chen , H. K. Kim , S. Zhang , C. Thai , R. D. McGillicuddy , H. Yan , J. J. Vlassak , J. A. Mason , J. Am. Chem. Soc. 2024, 146, 2736. 38227768 10.1021/jacs.3c12402

[27] A. Aznar , P. Lloveras , M. Romanini , M. Barrio , J.‐L. Tamarit , C. Cazorla , D. Errandonea , N. D. Mathur , A. Planes , X. Moya , L. Mañosa , Nat. Commun. 2017, 8, 1851. 29184055 10.1038/s41467-017-01898-2 PMC5705726

[28] A. K. Sagotra , D. Errandonea , C. Cazorla , Nat. Commun. 2017, 8, 963. 29042557 10.1038/s41467-017-01081-7 PMC5645463

[29] A. K. Sagotra , D. Chu , C. Cazorla , Nat. Commun. 2018, 9, 3337. 30127398 10.1038/s41467-018-05835-9 PMC6102246

[30] J. Min , A. K. Sagotra , C. Cazorla , Phys. Rev. Mater. 2020, 4, 015403.

[31] M. McLinden , Chapter 20, Thermophysical Properties of Refrigerants, ASHRAE Handbook: Fundamentals, 2005.

[32] M. Romanini , Y. Wang , K. Gürpinar , G. Ornelas , P. Lloveras , Y. Zhang , W. Zheng , M. Barrio , A. Aznar , A. Gràcia‐Condal , B. Emre , O. Atakol , C. Popescu , H. Zhang , Y. Long , L. Balicas , J. Lluís Tamarit , A. Planes , M. Shatruk , L. Mañosa , Adv. Mater. 2021, 33, 2008076. 10.1002/adma.202008076 33527567

[33] F. B. Li , M. Li , X. Xu , Z. C. Yang , H. Xu , C. K. Jia , K. Li , J. He , B. Li , H. Wang , Nat. Commun. 2020, 11, 4190. 32826887 10.1038/s41467-020-18043-1 PMC7442785

[34] F. Li , M. Li , C. Niu , H. Wang , Appl. Phys. Lett. 2022, 120, 073902.

[35] N. de Oliveira , Acta Mater. 2023, 246, 118657.

[36] K. Sau , T. Ikeshoji , S. Takagi , S.‐i. Orimo , D. Errandonea , D. Chu , C. Cazorla , Sci. Rep. 2021, 11, 11915. 34099742 10.1038/s41598-021-91123-4 PMC8184963

[37] W. Tang , A. Unemoto , W. Zhou , V. Stavila , M. Matsuo , H. Wu , S.‐I. Orimo , T. Udovic , Energy Environ. Sci. 2015, 8, 3637. 26955398 10.1039/C5EE02941D PMC4778258

[38] M. Dimitrievska , P. Shea , K. E. Kweon , M. Bercx , J. B. Varley , W. S. Tang , A. V. Skripov , V. Stavila , T. J. Udovic , B. C. Wood , Adv. Energy Mater. 2018, 8, 1703422.

[39] H. Hagemann , CHIMIA 2019, 73, 868. 31753067 10.2533/chimia.2019.868

[40] A. V. Skripov , R. V. Skoryunov , A. V. Soloninin , O. A. Babanova , W. S. Tang , V. Stavila , T. J. Udovic , J. Phys. Chem. C 2015, 119, 26912.

[41] R. Mohtadi , S.‐i. Orimo , Nat. Rev. Mater. 2016, 2, 16091.

[42] M. Dimitrievska , H. Wu , V. Stavila , O. A. Babanova , R. V. Skoryunov , A. V. Soloninin , W. Zhou , B. A. Trump , M. S. Andersson , A. V. Skripov , T. J. Udovic , J. Phys. Chem. C 2020, 124, 17992. 10.1021/acs.jpcc.0c05038 PMC11167872 38868723

[43] S. Kim , H. Oguchi , N. Toyama , T. Sato , S. Takagi , T. Otomo , D. Arunkumar , N. Kuwata , J. Kawamura , S.‐i. Orimo , Nat. Commun. 2019, 10, 1081. 30842419 10.1038/s41467-019-09061-9 PMC6403359

[44] S. Kim , K. Kisu , S. Takagi , H. Oguchi , S.‐I. Orimo , ACS Appl. Energy Mater. 2020, 3, 4831.

[45] K. Sau , T. Ikeshoji , S. Kim , S. Takagi , S. I. Orimo , Chem. Mater. 2021, 33, 2357.

[46] E. Stern‐Taulats , A. Gràcia‐Condal , A. Planes , P. Lloveras , M. Barrio , J.‐L. Tamarit , S. Pramanick , S. Majumdar , L. Mañosa , Appl. Phys. Lett. 2015, 107, 152409.

[47] L. Mañosa , D. González‐Alonso , A. Planes , M. Barrio , J.‐L. Tamarit , I. S. Titov , M. Acet , A. Bhattacharyya , S. Majumdar , Nat. Commun. 2011, 2, 595. 22186891 10.1038/ncomms1606

[48] D. Matsunami , A. Fujita , K. Takenaka , M. Kano , Nat. Mater. 2015, 14, 73. 25344781 10.1038/nmat4117

[49] N. M. Bom , W. Imamura , E. O. Usuda , L. S. Paixão , A. M. G. Carvalho , ACS Macro Lett. 2018, 7, 31. 35610934 10.1021/acsmacrolett.7b00744

[50] E. O. Usuda , W. Imamura , N. M. Bom , L. S. Paixão , A. M. G. Carvalho , ACS Appl. Polym. Mater. 2019, 1, 1991.

[51] A. Aznar , P. Lloveras , M. Barrio , P. Negrier , A. Planes , L. Mañosa , N. D. Mathur , X. Moya , J.‐L. Tamarit , J. Mater. Chem. A 2020, 8, 639.

[52] K. Zhang , R. Song , J. Qi , Z. Zhang , Z. Zhang , C. Yu , K. Li , Z. Zhang , B. Li , Adv. Funct. Mater. 2022, 32, 2112622.

[53] A. Aznar , P. Negrier , A. Planes , L. Manosa , E. Stern‐Taulats , X. Moya , M. Barrio , J.‐L. Tamarit , P. Lloveras , Appl. Mater. Today 2021, 23, 101023.

[54] W. Imamura , É. O. Usuda , L. S. Paixão , N. M. Bom , A. M. Gomes , A. M. G. Carvalho , Chin. J. Polym. Sci. 2020, 38, 999.

[55] J. Li , D. Dunstan , X. Lou , A. Planes , L. Mañosa , M. Barrio , J.‐L. Tamarit , P. Lloveras , J. Mater. Chem. A 2020, 8, 20354.

[56] J. Seo , J. D. Braun , V. M. Dev , J. A. Mason , J. Am. Chem. Soc. 2022, 144, 6493. 35360899 10.1021/jacs.2c01315

[57] J. M. Bermúdez‐García , M. Sánchez‐Andújar , S. Castro‐García , J. López‐Beceiro , R. Artiaga , M. A. Señarís‐Rodríguez , Nat. Commun. 2017, 8, 15715. 28569842 10.1038/ncomms15715 PMC5461497

[58] J. M. Bermúdez‐García , S. Yanez‐Vilar , A. Garcia‐Fernandez , M. Sanchez‐Andujar , S. Castro‐Garcia , J. Lopez‐Beceiro , R. Artiaga , M. Dilshad , X. Moya , M. A. Señarís‐Rodríguez , J. Mater. Chem. C 2018, 6, 9867.

[59] Q. Ren , J. Qi , D. Yu , Z. Zhang , R. Song , W. Song , B. Yuan , T. Wang , W. Ren , Z. Zhang , et al., Nat. Commun. 2022, 13, 2293. 35484158 10.1038/s41467-022-29997-9 PMC9051211

[60] E. Usuda , W. Imamura , N. Bom , L. Paixão , A. Carvalho , ACS Appl. Polym. Mater. 2019, 1, 1991.

[61] A. Aznar , A. Gràcia‐Condal , A. Planes , P. Lloveras , M. Barrio , J.‐L. Tamarit , W. Xiong , D. Cong , C. Popescu , L. Mañosa , Phys. Rev. Mater. 2019, 3, 044406.

[62] L. Mañosa , D. González‐Alonso , A. Planes , E. Bonnot , M. Barrio , J.‐L. Tamarit , S. Aksoy , M. Acet , Nat. Mater. 2010, 9, 478. 20364140 10.1038/nmat2731

[63] Z. Wei , Y. Shen , Z. Zhang , J. Guo , B. Li , E. Liu , Z. Zhang , J. Liu , APL Mater. 2020, 8, 051101.

[64] X. He , Y. Kang , S. Wei , Y. Zhang , Y. Cao , K. Xu , Z. Li , C. Jing , Z. Li , J. Alloys Compd. 2018, 741, 821.

[65] E. Stern‐Taulats , A. Planes , P. Lloveras , M. Barrio , J.‐L. Tamarit , S. Pramanick , S. Majumdar , C. Frontera , L. Mañosa , Phys. Rev. B 2014, 89, 214105.

[66] E. Stern‐Taulats , A. Gràcia‐Condal , A. Planes , P. Lloveras , M. Barrio , J.‐L. Tamarit , S. Pramanick , S. Majumdar , L. Mañosa , Appl. Phys. Lett. 2015, 107, 152409.

[67] A. Aznar , P. Lloveras , J.‐Y. Kim , E. Stern‐Taulats , M. Barrio , J. L. Tamarit , C. F. Sánchez‐Valdés , J. L. Sanchez Llamazares , N. D. Mathur , X. Moya , Adv. Mater. 2019, 31, 1903577. 10.1002/adma.201903577 31385369

[68] T. Samanta , P. Lloveras , A. Us Saleheen , D. L. Lepkowski , E. Kramer , I. Dubenko , P. W. Adams , D. P. Young , M. Barrio , J. L. Tamarit , et al., Appl. Phys. Lett. 2018, 112, 021907.

[69] P. Lloveras , T. Samanta , M. Barrio , I. Dubenko , N. Ali , J.‐L. Tamarit , S. Stadler , APL Mater. 2019, 7, 061106.

[70] H. Liu , Z. Li , Y. Zhang , Z. Ni , K. Xu , Y. Liu , Scr. Mater. 2020, 177, 1.

[71] E. Stern‐Taulats , P. Lloveras , M. Barrio , E. Defay , M. Egilmez , A. Planes , J.‐L. Tamarit , L. Mañosa , N. Mathur , X. Moya , APL Mater. 2016, 4, 091102.

[72] P. Lloveras , E. Stern‐Taulats , M. Barrio , J.‐L. Tamarit , S. Crossley , W. Li , V. Pomjakushin , A. Planes , L. Mañosa , N. Mathur , et al., Nat. Commun. 2015, 6, 8801. 26607989 10.1038/ncomms9801 PMC4674762

[73] A. Aznar , P. Lloveras , M. Romanini , M. Barrio , J.‐L. Tamarit , C. Cazorla , D. Errandonea , N. D. Mathur , A. Planes , X. Moya , et al., Nat. Commun. 2017, 8, 1851. 29184055 10.1038/s41467-017-01898-2 PMC5705726

[74] A. K. Sagotra , D. Chu , C. Cazorla , Nat. Commun. 2018, 9, 3337. 30127398 10.1038/s41467-018-05835-9 PMC6102246

[75] J. Min , A. K. Sagotra , C. Cazorla , Phys. Rev. Mater. 2020, 4, 015403.

[76] C. Escorihuela‐Sayalero , L. C. Pardo , M. Romanini , N. Obrecht , S. Loehlé , P. Lloveras , J.‐L. Tamarit , C. Cazorla , npj Comput. Mater. 2024, 10, 13.

[77] B. E. Meijer , R. J. C. Dixey , F. Demmel , R. Perry , H. C. Walker , A. E. Phillips , Phys. Chem. Chem. Phys. 2023, 25, 9282. 36919868 10.1039/d2cp05412d

[78] S. Yuan , B. E. Meijer , G. Cai , R. J. C. Dixey , F. Demmel , M. T. Dove , J. Liu , H. Y. Playford , H. C. Walker , A. E. Phillips , Adv. Funct. Mater. 2022, 32, 2207717.

[79] Z. Zhang , L. F. Nazar , Nat. Rev. Mater. 2022, 7, 389.

[80] S. Plimpton , J. Comput. Phys. 1995, 117, 1.

[81] G. Kresse , J. Hafner , Phys. Rev. B 1993, 47, 558. 10.1103/physrevb.47.558 10004490

[82] J. Perdew , K. Burke , M. Ernzerhof , Phys. Rev. Lett. 1996, 77, 3865. 10062328 10.1103/PhysRevLett.77.3865

[83] P. Blöchl , Phys. Rev. B 1994, 50, 17953. 10.1103/physrevb.50.17953 9976227

[84] A. K. Sagotra , D. Chu , C. Cazorla , Phys. Rev. Mater. 2019, 3, 035405.

[85] A. Togo , L. Chaput , I. Tanaka , G. Hug , Phys. Rev. B 2010, 81, 174301.

[86] S. Takagi , T. Ikeshoji , T. Sato , S.‐i. Orimo , Appl. Phys. Lett. 2020, 116, 173901.

