# Supplementary material for: Colossal Reversible Barocaloric Effects in a Plastic Crystal Mediated by Lattice Vibrations and Ion Diffusion
Source: Adv Sci (Weinh). 2024 May 5;11(26):2306488. doi: 10.1002/advs.202306488 (PMC11234397; doi:10.1002/advs.202306488)
Supplement: Supplementary file 1 — Supporting Information [file ADVS-11-2306488-s001.pdf]

ADVANCED SCIENCE
Open Access

Supporting Information

for *Adv. Sci.*, DOI 10.1002/advs.202306488

Colossal Reversible Barocaloric Effects in a Plastic Crystal Mediated by Lattice Vibrations and Ion Diffusion

Ming Zeng, Carlos Escorihuela-Sayalero, Tamio Ikeshoji, Shigeyuki Takagi, Sangryun Kim, Shin-ichi Orimo, María Barrio, Josep-Lluís Tamarit, Pol Lloveras, Claudio Cazorla* and Kartik Sau**

Supplementary Information for "Colossal reversible barocaloric effects in a plastic crystal mediated by lattice vibrations and ion diffusion"

Ming Zeng,[1] Carlos Escorihuela-Sayalero,[1] Tamio Ikeshoji,[2] Shigeyuki Takagi,[3] Sangryun Kim,[4] Shin-ichi Orimo,[5,3] María Barrio,[1] Josep-Lluís Tamarit,[1] Pol Lloveras,[1,*] Claudio Cazorla,[1,†] and Kartik Sau[5,2,‡]

[1]*Grup de Caracterització de Materials, Departament de Física, EEBE and Barcelona Research Center in Multiscale Science and Engineering Universitat Politècnica de Catalunya, Av. Eduard Maristany 10-14, Barcelona 08019, Catalonia, Spain*

[2]*Mathematics for Advanced Materials Open Innovation Laboratory (MathAM-OIL), National Institute of Advanced Industrial Science and Technology (AIST), c/o Advanced Institute for Materials Research (AIMR), Tohoku University, Sendai 980-8577, Japan*

[3]*Institute for Materials Research (IMR), Tohoku University, Sendai 980-8577, Japan*

[4]*Graduate School of Energy Convergence, Gwangju Institute of Science and Technology (GIST), 123 Cheomdangwagi-ro, Buk-gu, Gwangju 61005, Republic of Korea*

[5]*Advanced Institute for Materials Research (AIMR), Tohoku University, Sendai 980-8577, Japan*

The list of supplementary figures and tables in this document are the following:

- Figure S1: Powder X-ray diffraction (PXRD) experiments.
- Figure S2: Differential scanning calorimetry (DSC) experiments.
- Figure S3: Experimental heat capacity at atmospheric pressure.
- Figure S4: Experimental entropy curves, $S(T, p)$.
- Figure S5: Pre-transitional effects in the molecular dynamics (MD) simulations.
- Figure S6: Vibrational density of states (VDOS) obtained from MD simulations.
- Figure S7: VDOS obtained from MD and *ab initio* molecular dynamics (AIMD) simulations.
- Figure S8: Results of constrained MD simulations.
- Figure S9: Results of convergence tests for estimation of thermodynamic properties.
- Table SI: Summary of materials for which reversible barocaloric effects have been reported.
- Table SII: Interatomic potential parameters employed in this study.

* pol.lloveras@upc.edu

† claudio.cazorla@upc.edu

‡ kartik.sau@gmail.com

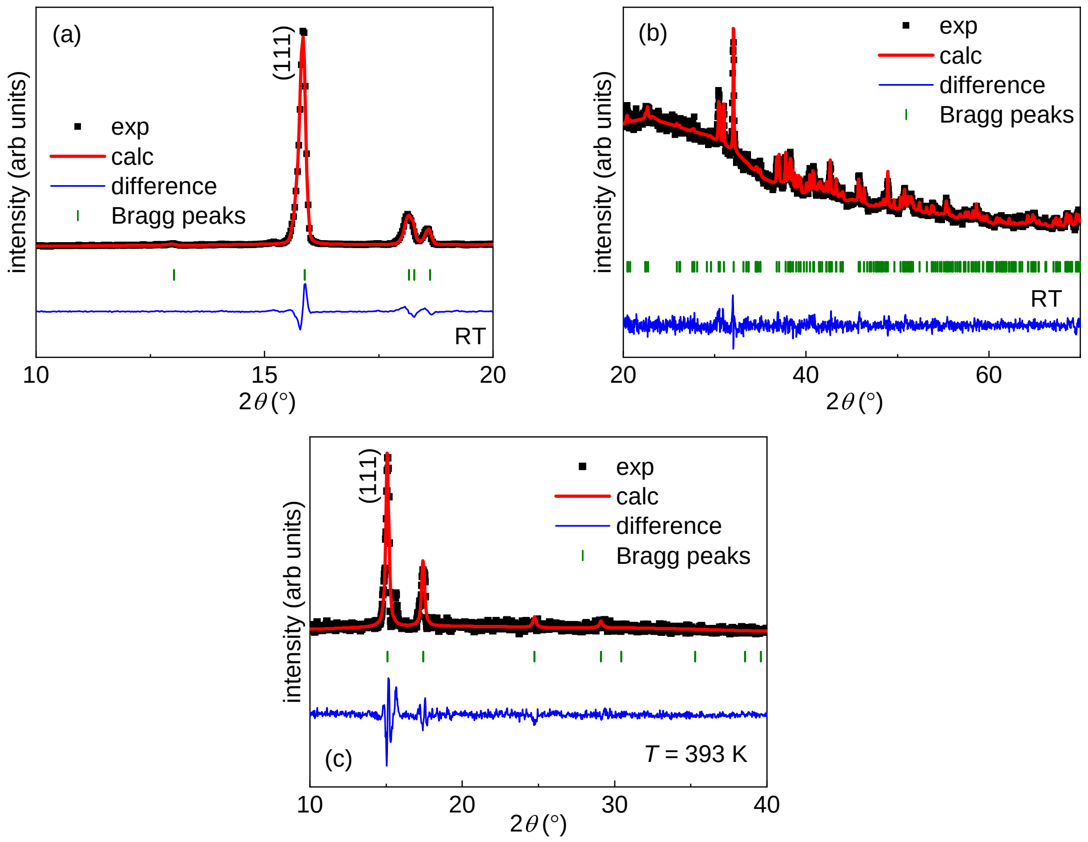

FIG. S1. Powder X-ray diffraction (PXRD) was carried out on a transmission mode diffractometer using Debye-Scherrer geometry equipped with cylindrical position sensitive detectors (CPS120) from INEL (France) containing 4096 channels (0.029° 2θ angular step) with monochromatic Cu $K_{\alpha 1}$ (λ = 1.5406 Å) radiation. External calibration using the $Na_2Ca_2Al_2F_{14}$ cubic phase was performed by means of pseudo-voigt fits. The system was equipped with a liquid nitrogen 700 series Cryostream Cooler (Oxford Cryosystems) with an accuracy of 0.1 K. The sample was introduced in a Lindemann capillary (0.5 mm diameter) and subjected to high vacuum at 493 K for several days before the PXRD experiments. Measurements between 200 and 400K were performed on heating with acquisition times of one hour after temperature stabilization of 15 minutes. (a)-(b) Room-temperature results. (c) High-temperature results.

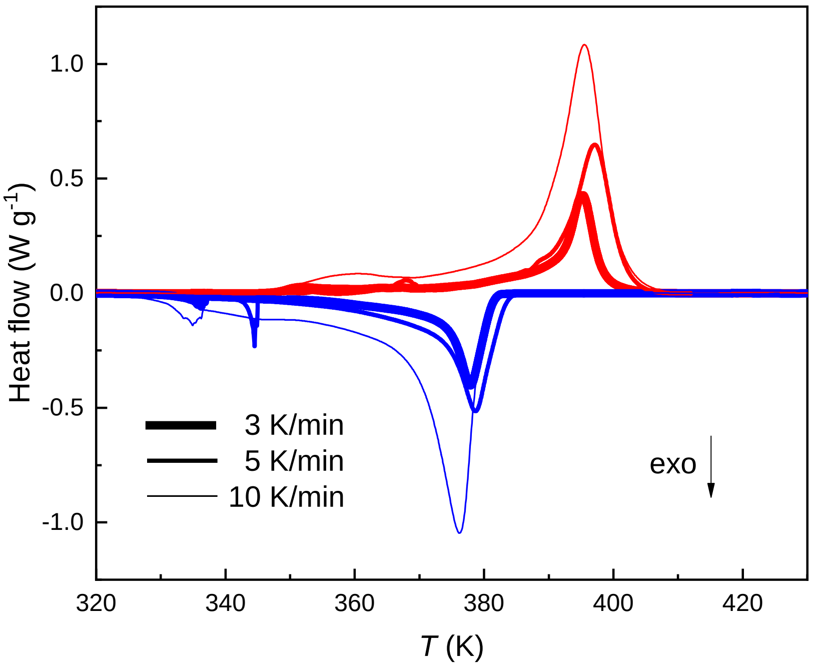

FIG. S2. Differential scanning calorimetry (DSC) performed on $LiCB_{11}H_{12}$ at atmospheric pressure for different scanning rates. The small peaks appreciated in the figure are due to sample inhomogeneities. The mass of the samples was ≈ 10 mg and these were hermetically encapsulated in Aluminum pans.

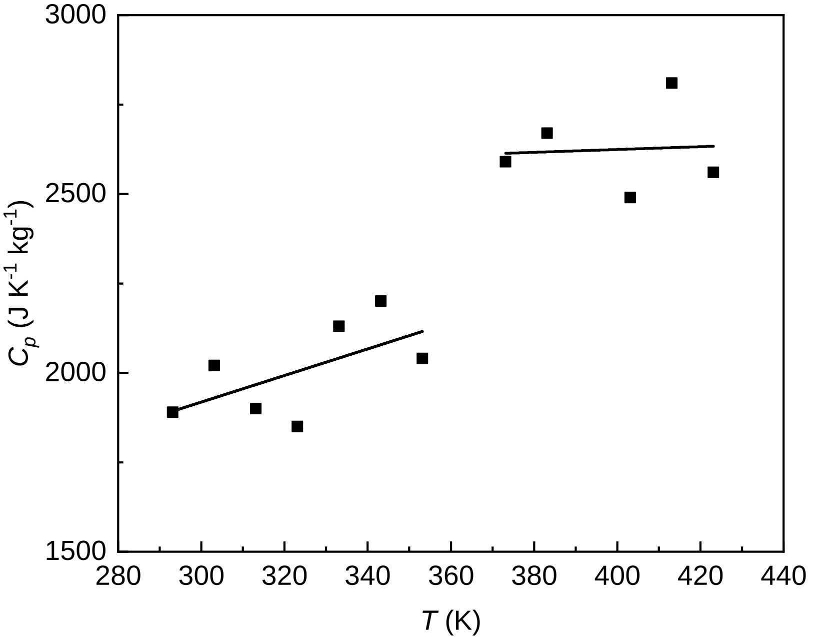

FIG. S3. Heat capacity of $LiCB_{11}H_{12}$ at atmospheric pressure measured by modulated differential scanning calorimetry in isothermal conditions. Straight lines are fits to the data.

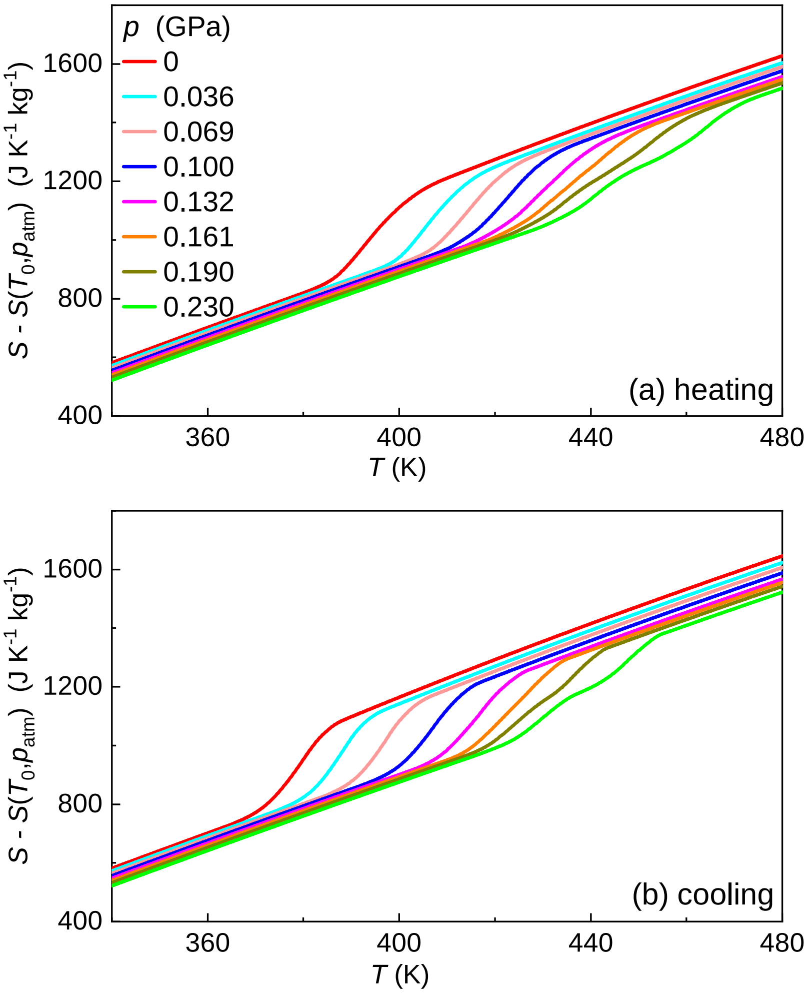

FIG. S4. Entropy as a function of temperature at different pressures, $S(T,p)$, determined on (a) heating and (b) on cooling, with respect to the reference temperature T_0 and $p_{\rm atm}$. For more details, see the Methods section in the main manuscript.

Chemical formula (name/acronym)	$\lvert\Delta S_{rev}\rvert$ (J K^{-1} kg^{-1})	$\lvert\Delta T_{rev}\rvert$ (K)	$\frac{\lvert\Delta S_{rev}\rvert}{\Delta p}$ (J K^{-1} kg^{-1} MPa^{-1})	Δp (MPa)	Ref.
$LiCB_{11}H_{12}$	62.7	3.9	0.905	69	This work
$LiCB_{11}H_{12}$	131	6.9	1.532	86	This work
$LiCB_{11}H_{12}$	180	10.5	1.787	101	This work
$LiCB_{11}H_{12}$	220	14.3	1.887	117	This work
$LiCB_{11}H_{12}$	240	17.6	1.812	133	This work
$LiCB_{11}H_{12}$	252	20.2	1.708	148	This work
$LiCB_{11}H_{12}$	261	22.8	1.622	161	This work
$LiCB_{11}H_{12}$	267	25.3	1.504	177	This work
$LiCB_{11}H_{12}$	271	27.0	1.421	190	This work
$LiCB_{11}H_{12}$	277	29.9	1.306	212	This work
$LiCB_{11}H_{12}$	283	31.9	1.236	229	This work
$C_5H_{12}O_2$ (neopentylglycol)	400	8.2	1.581	253	[S1]
$C_5H_{12}O_3$ (pentaglycerine)	385	4.7	2.404	160	[S1]
$C_5H_{12}O$ (neoptenylalcohol)	290	16	1.115	260	[S1]
$C_{10}H_{15}Br$ (1-bromoadamantane)	140.4	20	1.404	100	[S2]
$C_{10}H_{15}Cl$ (1-chloroadamantane)	162.9	16	1.629	100	[S2]
$C_{10}H_{16}O$ (1-adamantanol)	175	11	1.094	160	[S3]
$C_{10}H_{16}O$ (2-adamantanol)	76.3	7.4	0.587	130	[S3]
$C_{10}H_{16}O$ (2-adamantanol)	100	8	0.541	185	[S3]
$C_{11}H_{18}O$ (2-methyl-2-adamantanol)	300	7	3.75	80	[S3]
$C_2B_{10}H_{12}$ (orthocarborane)	57	14	1.910	30	[S4]
$C_2B_{10}H_{12}$ (orthocarborane)	79	14	1.58	50	[S4]
$C_2B_{10}H_{12}$ (metacarborane)	71	13	1.42	50	[S4]
$C_2B_{10}H_{12}$ (paracarborane)	97	19	1.94	50	[S4]
C_{60}	30	9.7	0.3	100	[S5]
$Fe_3(bntrz)_6(tcnset)_6$	80	10	1.455	55	[S6]
$Fe_3(bntrz)_6(tcnset)_6$	120	35	0.462	260	[S6]
$Fe[HB(tz)_3]_2$	92	2.1	6.133	150	[S7]
$(C_{10}H_{21}NH_3)_2MnCl_4$	230	5	4.6	50	[S5]
$(C_{10}H_{21}NH_3)_2MnCl_4$	250	12	2.5	100	[S5]
$(C_{10}H_{21}NH_3)_2MnCl_4$	248	6.9	4.96	50	[S8]
$[TPrA]Mn[dca]_3$	30.5	4.1	4.357	7	[S9]
$[TPrA]Cd[dca]_3$	11.5	1.4	1.643	7	[S10]
$(NA)_2CuBr_4$	91.3	9.4	1.826	50	[S8]
NH_4I	71	34	1.775	40	[S11]
Acetoxy Silicone Rubber	182	21	1.052	173	[S12]
Nitrile Butadiene Rubber	59	16.4	0.151	0.39	[S13]
$Ni_{50}Mn_{31.5}Ti_{18.5}$	35	3.7	0.092	380	[S14]
$Ni_{49.26}Mn_{36.08}In_{14.66}$	24	4.5	0.092	260	[S15]
$Ni_{35.5}Co_{14.5}Mn_{35}Ti_{15}$	8.5	2.3	0.085	100	[S16]
$Ni_{42.3}Co_{7.9}Mn_{38.8}Sn_{11.0}$	15	5	0.024	620	[S17]

(Continuation)

Chemical formula (name/acronym)	$\|\Delta S_{\rm rev}\|$ (J K^{-1} kg^{-1})	$\|\Delta T_{\rm rev}\|$ (K)	$\frac{\|\Delta S_{\rm rev}\|}{\Delta p}$ (J K^{-1} kg^{-1} MPa^{-1})	Δp (MPa)	Ref.
$Fe_{49}Rh_{51}$	12	5	0.048	250	[S18, S19]
$MnCoGeB_{0.03}$	30	12.5	0.115	260	[S20]
$(MnNiSi)_{0.62}(FeCoGe)_{0.38}$	74	16	0.274	270	[S21]
$(MnNiSi)_{0.61}(FeCoGe)_{0.39}$	44	6	0.169	260	[S22]
$(MnNiSi)_{0.60}(FeCoGe)_{0.40}$	47	4	0.204	230	[S22]
$(MnNiSi)_{0.59}(FeCoGe)_{0.41}$	24	1.8	0.104	230	[S22]
$Co_{50}Fe_{2.5}V_{31.5}Ga_{16}$	31	6	0.062	500	[S23]
$BaTiO_3$	1.47	4	0.0147	100	[S24]
$(NH_4)_2SO_4$	58	9.4	0.232	250	[S25]
AgI	60	18	0.24	250	[S26]
Li_3N	32	2.8	0.032	1000	[S27]
Cu_2Se	17.5	8.7	0.0175	1000	[S28]

TABLE SI. Summary of materials for which reversible barocaloric effects have been reported. The reversible barocaloric strength is defined as $|\Delta S_{\rm rev}/\Delta p|$, where Δp refers to the pressure at which the reversible barocaloric effects $|\Delta S_{\rm rev}|$ and $|\Delta T_{\rm rev}|$ were measured. Values for each material have been selected as those that best illustrate the overall barocaloric performance, according to the criteria of the authors.

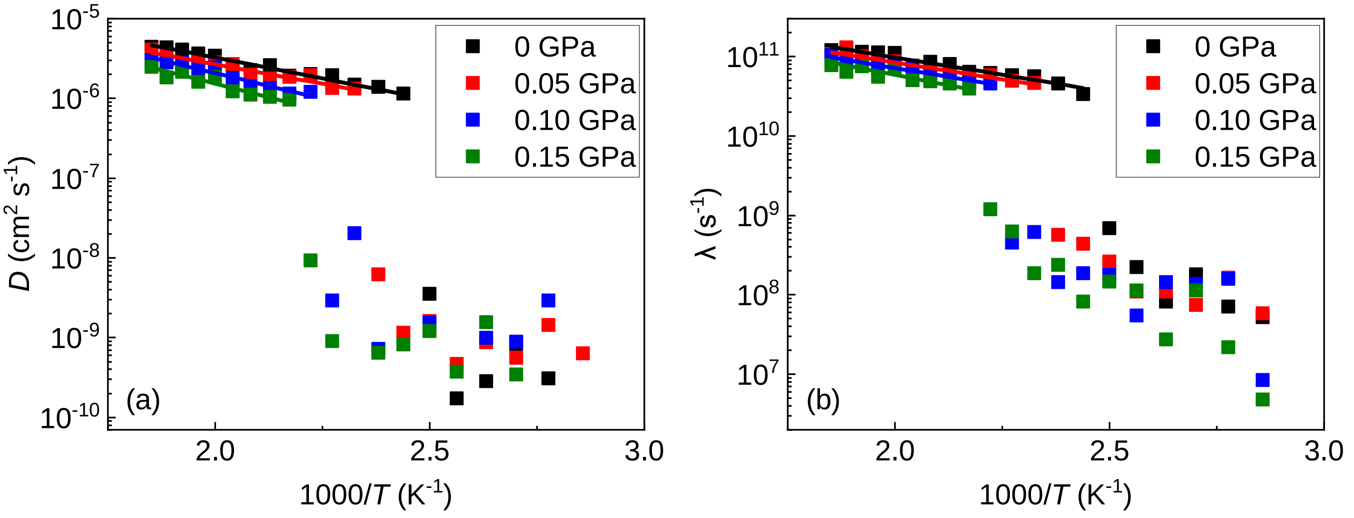

FIG. S5. Pre-transitional effects in molecular dynamics (MD) simulations of bulk $LiCB_{11}H_{12}$. Upon increasing pressure, the diffusion coefficient of "slow" lithium ions in the ordered phase slightly increases (a). Molecular orientational motion in the ordered phase is negligible and independent of pressure (b).

Species (M)	q_M	$A_{M-\mathrm{Li}}$	$A_{M-\mathrm{B}}$	$A_{M-\mathrm{H}}$	$\rho_{M-\mathrm{Li}}$	$\rho_{M-\mathrm{B}}$	$\rho_{M-\mathrm{H}}$
	(e)	(eV)	(eV)	(eV)	(Å)	(Å)	(Å)
Li	0.75	0.0	46.08	76.4	0.4000	0.2853	0.3023
C	0.75	46.08	0.0	0.0	0.2853	0.0	0.2853
B	0.0	80.78	0.0	0.0	0.2853	0.0	0.2853
H	-0.125	76.4	0.0	2175.5	0.3023	0.2853	0.20855

TABLE SII. Interatomic potential parameters employed in this study [S29]. k_{BB}= 5.375 Å^{-2}eV, r_{BB0} = 1.76 Å (equilibrium bond length), $k_{\mathrm{C-B}}$ = 5.375 Å^{-2}eV, r_{CB0} = 1.76 Å (equilibrium bond length), $k_{\mathrm{B-H}}$ = 9.89 Å^{-2}eV, r_{BH0} = 1.2 Å (equilibrium bond length), $k_{\mathrm{C-H}}$ = 150.5 Å^{-2}eV, r_{CH0} = 1.09 Å (equilibrium bond length), $k_{\mathrm{B-B-H}} = k_{\mathrm{B-C-H}} = k_{\mathrm{C-B-H}}$ = 0.86 rad^{-2}eV, θ_0 = 2.077 rad (equilibrium angle), $C_{\mathrm{H-H}}$ = 4.3086 Å^{6}eV.

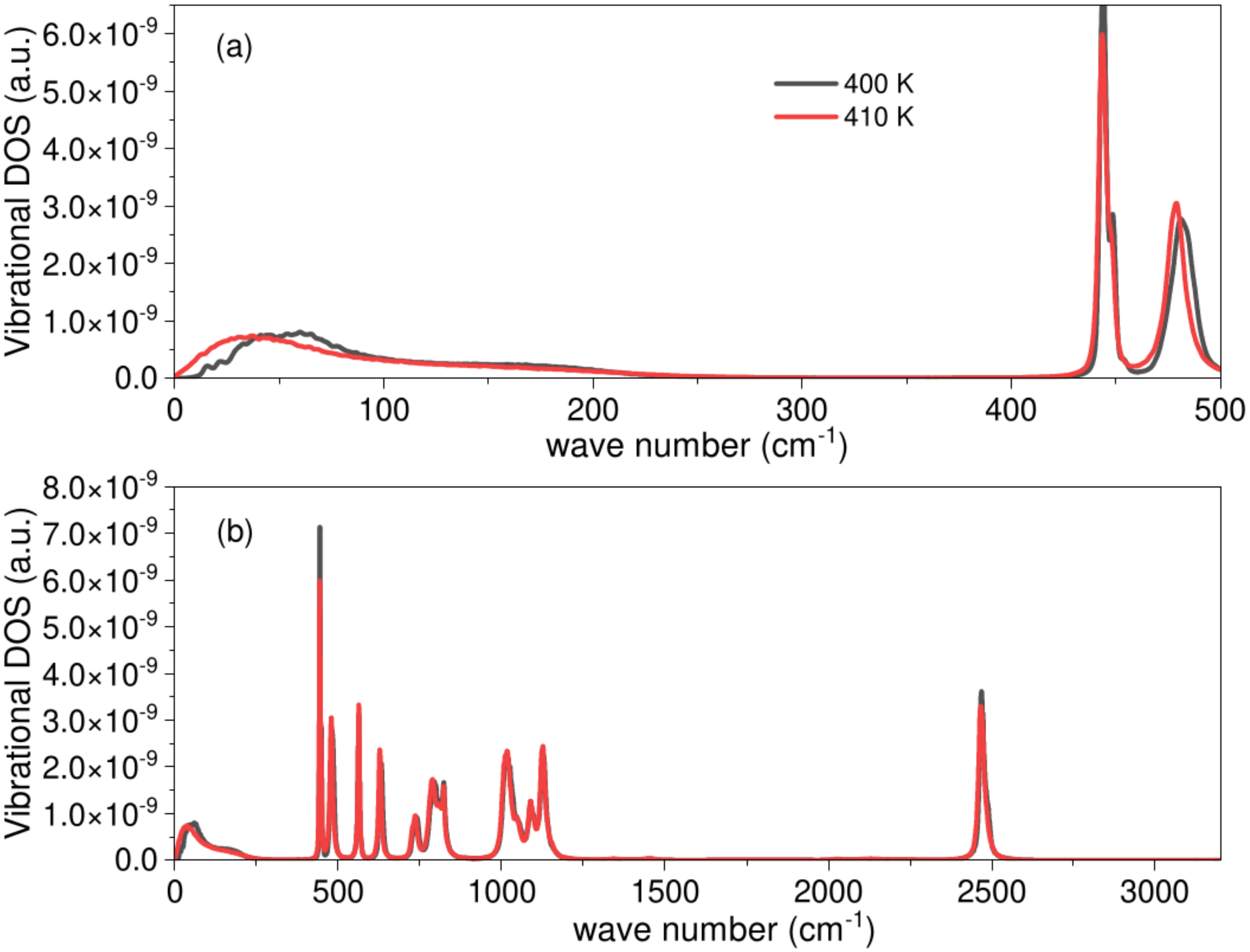

FIG. S6. Vibrational density of states (VDOS) of bulk $LiCB_{11}H_{12}$ estimated from molecular dynamics (MD) simulations for the ordered and disordered phases in the (a) low-frequency and (b) full frequency ranges. Vibrational modes with frequencies lower than ≈ 300 cm^{-1} correspond to libration and inter-molecule excitations while higher frequencies to intra-molecular modes (e.g., bonds stretching). The high-T disordered phase presents a larger number of low-frequency vibrational modes (e.g., < 50 cm^{-1}) than the low-T ordered phase, thus explaining its larger vibrational entropy near room temperature.

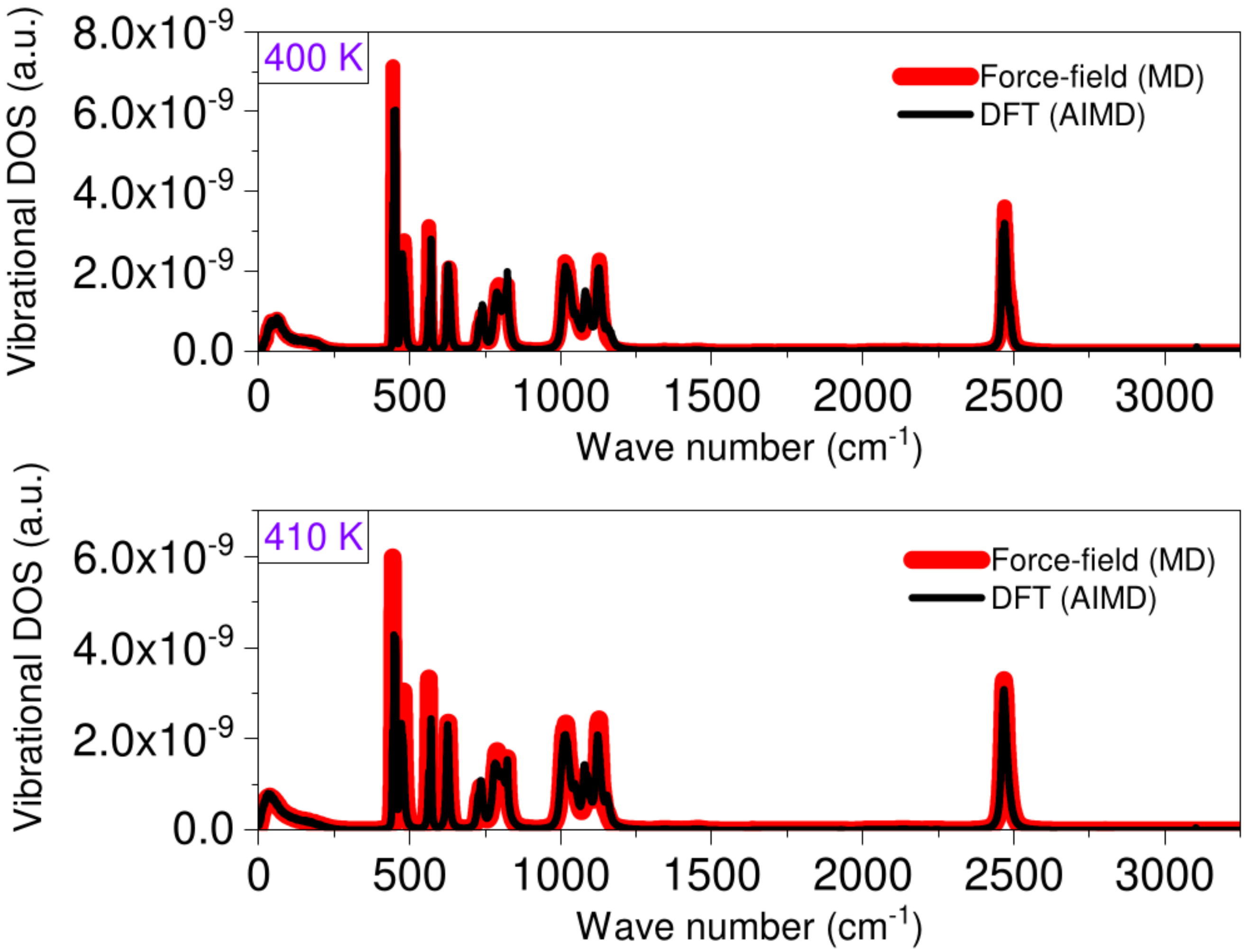

FIG. S7. Comparison of the vibrational density of states (VDOS) obtained for bulk $LiCB_{11}H_{12}$ from molecular dynamics (MD) simulations based on force fields and *ab initio* molecular dynamics (AIMD) simulations based on density functional theory (DFT). The agreement between the two sets of data is excellent. Details of the AIMD simulations can be found in the Methods section in the main text.

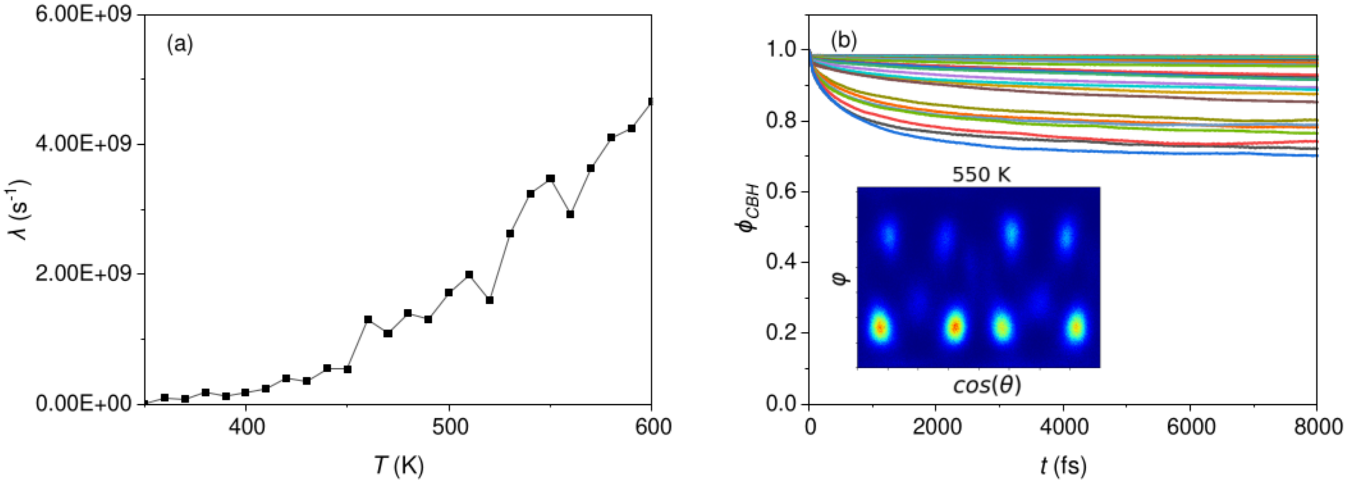

FIG. S8. Results of constrained molecular dynamics (MD) simulations performed at zero pressure. (a) Molecular orientational frequency and (b) angular autocorrelation function obtained from constrained MD simulations in which the lithium ions were pinned to their equilibrium positions. Molecular orientational disorder becomes apparent at temperatures > 550 K, that is, well above the phase transition temperature determined in the unconstrained MD simulations (i.e., $T_t \approx 400$ K). The explored temperature range was $350 \leq T \leq 600$ K and the simulations were performed at intervals of 10 K.

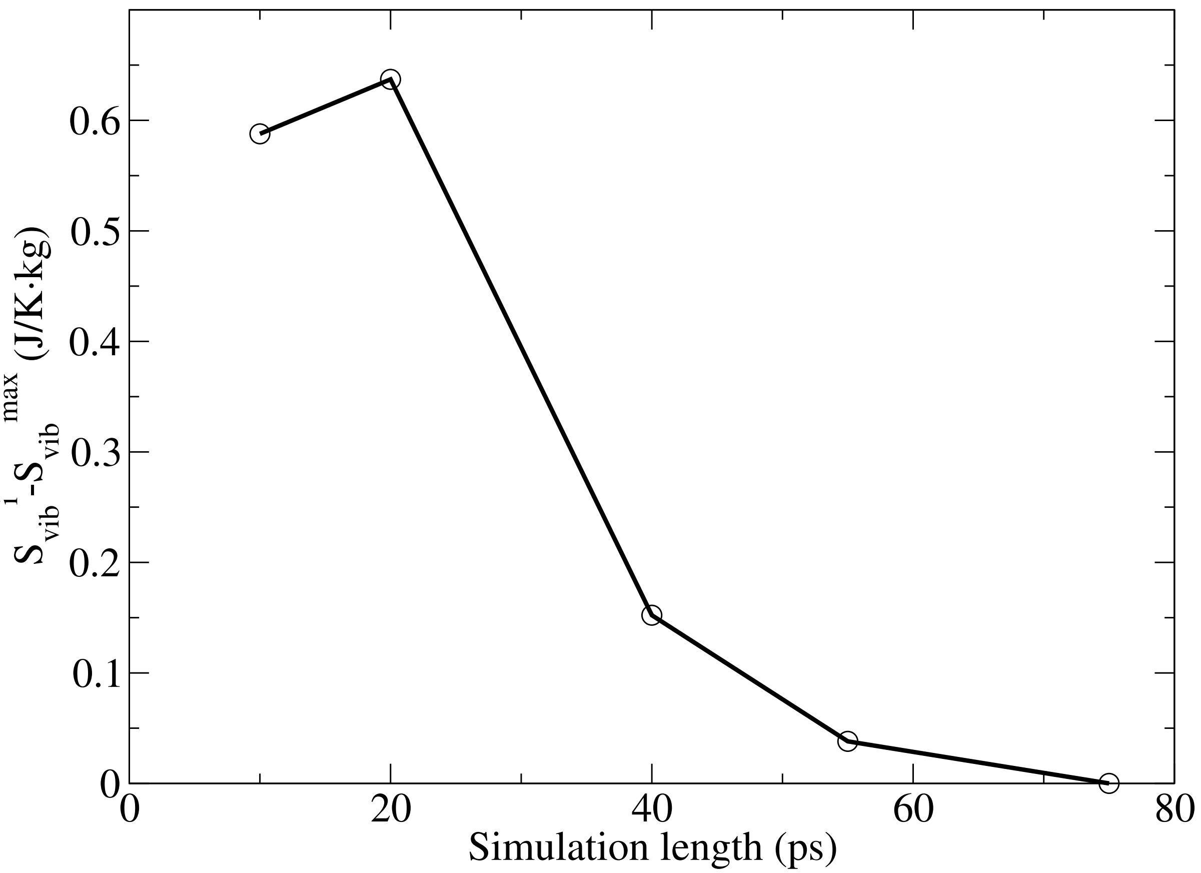

FIG. S9. Convergence of the vibrational entropy calculation as a function of the simulation length for a simulation at 400 K of LCBH. We define S^i_{vib} as the entropy calculated using a simulation length of i ps, and S^{max}_{vib} the entropy calculated using the maximum precision considered for the convergence study (75 ps).

SUPPLEMENTARY DESCRIPTION OF LCBH

Thermal conductivity. Specific information regarding the thermal conductivity of LCBH is currently unavailable. However, it is worth noting that boron-based compounds generally exhibit relatively low thermal conductivities as compared to metals and other inorganic materials. This characteristic can be attributed to their unique crystal structures and the presence of light elements such as boron and hydrogen, which typically possess lower thermal conductivities.

Cost. LCBH is a relatively expensive material, priced at 5,250 USD for 10 grams with a declared chemical purity of over 98%. In comparison, other materials without isotopes, such as $Li_2B_{12}H_{12}$ (545 USD for 10 grams) and $C_2B_{10}H_{12}$ (507 USD for 10 grams), are relatively cheaper.

Thermal and chemical stability. LCBH exhibits good thermal stability below 300 °C and chemical stability as well. However, it has a tendency to absorb moisture from the air. Fortunately, this moisture can be easily eliminated through heat treatment.

Processability. Complex metal hydrides like LCBH can be processed into various forms, including powders, pellets, films and composites. Several methods, such as ball milling, hot pressing, pulsed laser deposition and melt spinning, can be employed for this processing. Additionally, this material is soluble in water.

Tunability. Complex metal hydrides offer a high degree of tunability in terms of composition, structure, and properties. Cation exchange is a relatively simple process; however, modifying the anions is more challenging.

Scalability. At present, LCBH has not been commercialized and is not available in large-scale production.

[S1] A. Aznar, P. Lloveras, M. Barrio, P. Negrier, A. Planes, L. Mañosa, N. D. Mathur, X. Moya, and J.-L. Tamarit, Journal of materials chemistry A **8**, 639 (2020).
[S2] A. Aznar, P. Negrier, A. Planes, L. Manosa, E. Stern-Taulats, X. Moya, M. Barrio, J.-L. Tamarit, and P. Lloveras, Applied Materials Today **23**, 101023 (2021).
[S3] A. Salvatori, P. Negrier, A. Aznar, M. Barrio, J. L. Tamarit, and P. Lloveras, APL Materials **10**, 111117 (2022).
[S4] K. Zhang, R. Song, J. Qi, Z. Zhang, Z. Zhang, C. Yu, K. Li, Z. Zhang, and B. Li, Advanced Functional Materials **32**, 2112622 (2022).
[S5] J. Li, D. Dunstan, X. Lou, A. Planes, L. Mañosa, M. Barrio, J.-L. Tamarit, and P. Lloveras, Journal of Materials Chemistry A **8**, 20354 (2020).
[S6] M. Romanini, Y. Wang, K. Gürpinar, G. Ornelas, P. Lloveras, Y. Zhang, W. Zheng, M. Barrio, A. Aznar, A. Gràcia-Condal, *et al.*, Advanced Materials **33**, 2008076 (2021).
[S7] J. Seo, J. D. Braun, V. M. Dev, and J. A. Mason, Journal of the American Chemical Society **144**, 6493 (2022).
[S8] J. Seo, R. D. McGillicuddy, A. H. Slavney, S. Zhang, R. Ukani, A. A. Yakovenko, S.-L. Zheng, and J. A. Mason, Nature Communications **13**, 2536 (2022).
[S9] J. M. Bermúdez-García, M. Sánchez-Andújar, S. Castro-García, J. López-Beceiro, R. Artiaga, and M. A. Señarís-Rodríguez, Nature communications **8**, 15715 (2017).
[S10] J. M. Bermúdez-García, S. Yanez-Vilar, A. Garcia-Fernandez, M. Sanchez-Andujar, S. Castro-Garcia, J. Lopez-Beceiro, R. Artiaga, M. Dilshad, X. Moya, and M. A. Señarís-Rodríguez, Journal of Materials Chemistry C **6**, 9867 (2018).
[S11] Q. Ren, J. Qi, D. Yu, Z. Zhang, R. Song, W. Song, B. Yuan, T. Wang, W. Ren, Z. Zhang, *et al.*, Nature Communications **13**, 2293 (2022).
[S12] W. Imamura, É. O. Usuda, L. S. Paixão, N. M. Bom, A. M. Gomes, and A. M. G. Carvalho, Chinese Journal of Polymer Science **38**, 999 (2020).
[S13] E. Usuda, W. Imamura, N. Bom, L. Paixão, and A. Carvalho, ACS Applied Polymer Materials **1**, 1991 (2019).
[S14] A. Aznar, A. Gràcia-Condal, A. Planes, P. Lloveras, M. Barrio, J.-L. Tamarit, W. Xiong, D. Cong, C. Popescu, and L. Mañosa, Physical Review Materials **3**, 044406 (2019).
[S15] L. Mañosa, D. González-Alonso, A. Planes, E. Bonnot, M. Barrio, J.-L. Tamarit, S. Aksoy, and M. Acet, Nature materials **9**, 478 (2010).
[S16] Z. Wei, Y. Shen, Z. Zhang, J. Guo, B. Li, E. Liu, Z. Zhang, and J. Liu, APL Materials **8**, 051101 (2020).
[S17] X. He, Y. Kang, S. Wei, Y. Zhang, Y. Cao, K. Xu, Z. Li, C. Jing, and Z. Li, Journal of Alloys and Compounds **741**, 821 (2018).
[S18] E. Stern-Taulats, A. Planes, P. Lloveras, M. Barrio, J.-L. Tamarit, S. Pramanick, S. Majumdar, C. Frontera, and L. Mañosa, Physical Review B **89**, 214105 (2014).
[S19] E. Stern-Taulats, A. Gràcia-Condal, A. Planes, P. Lloveras, M. Barrio, J.-L. Tamarit, S. Pramanick, S. Majumdar, and L. Mañosa, Applied Physics Letters **107**, 152409 (2015).
[S20] A. Aznar, P. Lloveras, J.-Y. Kim, E. Stern-Taulats, M. Barrio, J. L. Tamarit, C. F. Sánchez-Valdés, J. L. Sanchez Llamazares, N. D. Mathur, and X. Moya, Advanced Materials **31**, 1903577 (2019).
[S21] T. Samanta, P. Lloveras, A. Us Saleheen, D. L. Lepkowski, E. Kramer, I. Dubenko, P. W. Adams, D. P. Young, M. Barrio, J. L. Tamarit, *et al.*, Applied Physics Letters **112**, 021907 (2018).
[S22] P. Lloveras, T. Samanta, M. Barrio, I. Dubenko, N. Ali, J.-L. Tamarit, and S. Stadler, APL Materials **7**, 061106 (2019).
[S23] H. Liu, Z. Li, Y. Zhang, Z. Ni, K. Xu, and Y. Liu, Scripta Materialia **177**, 1 (2020).
[S24] E. Stern-Taulats, P. Lloveras, M. Barrio, E. Defay, M. Egilmez, A. Planes, J.-L. Tamarit, L. Mañosa, N. Mathur, and X. Moya, APL Materials **4**, 091102 (2016).
[S25] P. Lloveras, E. Stern-Taulats, M. Barrio, J.-L. Tamarit, S. Crossley, W. Li, V. Pomjakushin, A. Planes, L. Mañosa, N. Mathur, *et al.*, Nature communications **6**, 8801 (2015).
[S26] A. Aznar, P. Lloveras, M. Romanini, M. Barrio, J.-L. Tamarit, C. Cazorla, D. Errandonea, N. D. Mathur, A. Planes, X. Moya, *et al.*, Nature communications **8**, 1851 (2017).
[S27] A. K. Sagotra, D. Chu, and C. Cazorla, Nature communications **9**, 3337 (2018).
[S28] J. Min, A. K. Sagotra, and C. Cazorla, Physical Review Materials **4**, 015403 (2020).
[S29] K. Sau, S. Takagi, T. Ikeshoji, K. Kisu, R. Sato, and S.-i. Orimo, Research Square (2022), 10.21203/rs.3.rs-1881421/v1.
